# Structure of the *Bacillus subtilis* 70S ribosome reveals the basis for species-specific stalling

**DOI:** 10.1038/ncomms7941

**Published:** 2015-04-23

**Authors:** Daniel Sohmen, Shinobu Chiba, Naomi Shimokawa-Chiba, C. Axel Innis, Otto Berninghausen, Roland Beckmann, Koreaki Ito, Daniel N. Wilson

**Affiliations:** 1 Gene Center and Department for Biochemistry, University of Munich, Feodor-Lynen-Street 25, Munich 81377, Germany; 2 Faculty of Life Sciences, Kyoto Sangyo University, Motoyama, Kamigamo, Kita-Ku, Kyoto 603-8555, Japan; 3 Institut Européen de Chimie et Biologie, Université de Bordeaux, Pessac, France; 4 Institut National de la Santé et de la Recherche Médicale (U869), Bordeaux, France; 5 Center for integrated Protein Science Munich (CiPSM), University of Munich, Feodor-Lynen-Street 25, Munich 81377, Germany

## Abstract

Ribosomal stalling is used to regulate gene expression and can occur in a species-specific manner. Stalling during translation of the MifM leader peptide regulates expression of the downstream membrane protein biogenesis factor YidC2 (YqjG) in *Bacillus subtilis*, but not in *Escherichia coli*. In the absence of structures of Gram-positive bacterial ribosomes, a molecular basis for species-specific stalling has remained unclear. Here we present the structure of a Gram-positive *B. subtilis* MifM-stalled 70S ribosome at 3.5–3.9 Å, revealing a network of interactions between MifM and the ribosomal tunnel, which stabilize a non-productive conformation of the PTC that prevents aminoacyl-tRNA accommodation and thereby induces translational arrest. Complementary genetic analyses identify a single amino acid within ribosomal protein L22 that dictates the species specificity of the stalling event. Such insights expand our understanding of how the synergism between the ribosome and the nascent chain is utilized to modulate the translatome in a species-specific manner.

## Abstract (web summary)

Ribosome stalling regulates gene expression by exposing otherwise inaccessible downstream ribosome-binding sites. Here the authors present a high-resolution Cryo-EM structure of the *Bacillus subtilis* MifM-stalled 70S ribosome to provide mechanistic insight into species-specific nascent peptide induced translational arrest.

## Graphical abstract

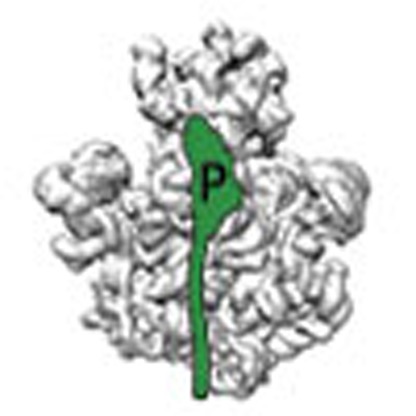

The ribosome is the major protein-synthesizing machine in the cell that converts the genetic information present within the codons of the mRNA into an amino-acid sequence within the polypeptide chain^1^. Ribosomes can transiently pause or even become stalled for prolonged periods during the translation of mRNAs due to many intrinsic factors, such as secondary structure within the mRNA^2^ or the presence of particular amino-acid sequences within the nascent polypeptide chain, such as polyproline sequences^3^,^4^. In many cases, ribosome stalling occurs during translation of short upstream open reading frames, so-called leader peptides, which is utilized by both bacteria and eukaryotes to regulate expression of downstream genes^5^,^6^. These regulatory events can be ligand-dependent, as exemplified by the ErmCL and TnaC leader peptides that require the presence of a macrolide antibiotic or free tryptophan, respectively, to induce ribosome stalling^7^,^8^, or ligand independent, with well-characterized examples including the *E. coli* SecM and *B. subtilis* MifM leader peptides, where translation of the amino-acid sequence is sufficient to induce the translational arrest^9^,^10^,^11^.

In *B. subtilis*, the MifM leader peptide is located upstream of the *yidC2* (*yqjG*) gene (Fig. 1a), which cannot be translated independently of MifM because the ribosome-binding site of *yidC2* is sequestered within a stem-loop structure in the mRNA (Fig. 1a)^10^. Ribosome stalling during translation of MifM leads to unwinding of the stem-loop structure and maintains the ribosome-binding site accessible for ribosome binding and induction of YidC2 expression (Fig. 1b). YidC2 is a homologue of the constitutively expressed SpoIIIJ (YidC1), a protein involved in membrane protein insertion and folding^12^. The *B. subtilis* MifM leader peptide is 95 amino-acids long and comprises a C-terminal region (residues 69–89) that is critical for ribosome stalling as well as an N-terminal transmembrane (TM) segment that targets the MifM peptide for membrane insertion, presumably via SpoIIIJ^10^. Interaction between SpoIIIJ and the TM segment of MifM as it emerges from the ribosomal tunnel is thought to prevent ribosome stalling by providing a pulling force on the MifM nascent chain^11^, analogous to SecA relief of SecM stalling^13^,^14^,^15^. Subsequently, canonical translation termination and ribosome-recycling ensues, leading to rapid refolding of the mRNA and repression of YidC2 expression (Fig. 1a). In contrast, when cellular levels of SpoIIIJ are low, ribosome stalling occurs on MifM, maintaining the unfolded conformation of the mRNA and thereby promoting expression of YidC2. In this manner, *B. subtilis* ensures that sufficient levels of SpoIIIJ or YidC2 are present in the membrane to direct membrane protein insertion and/or protein folding^10^,^16^.

Biochemical studies have demonstrated that four major ribosome-stalling sites are present in the MifM leader peptide; the first occurring when the codon for residue D89 is present in the P-site, then ribosomes stall at the following three codon positions corresponding to residues A90, G91 and S92 (Fig. 1b)^6^. Mutagenesis studies have identified six residues (R69, I70, W73, I74, M80 and N81) as well as the negatively charged DEED sequence (residues 86–89) within the C-terminal region of MifM (Fig. 1b) that are important for ribosome stalling^6^,^10^. Despite the high conservation of the ribosomal tunnel, translational stalling by MifM occurs on *B. subtilis* ribosomes, but not efficiently on *E. coli* ribosomes^11^; however, a molecular basis for this species specificity has remained unclear.

Here we have developed a *B. subtilis* cell-free *in vitro* translation system, which was subsequently used to generate a *B. subtilis* MifM-stalled ribosome complex (MifM-SRC). We present a cryoelectron microscopy (cryo-EM) reconstruction of the MifM-SRC at 3.5–3.9 Å resolution, which was used to build the first molecular model of a Gram-positive 70S ribosome. The only contacts of the MifM nascent chain with the ribosome that are not conserved between *B. subtilis* and *E. coli* are located within the lower region of the exit tunnel where they involve ribosomal protein L22. Consistently, our complementary genetic mutagenesis studies identified a single amino-acid residue, Met90 of L22, which modulates the specificity of MifM-dependent stalling. Interaction of the MifM nascent chain within the ribosomal tunnel positions the sidechain of Glu88 of MifM in a manner that prevents accommodation of an incoming aminoacyl-transfer RNA (tRNA) at the peptidyltransferase centre (PTC) of the ribosome, thus providing a structural basis for the MifM-dependent translational arrest.

## Results

### Cryo-EM structure of *B. subtilis* MifM-SRC

**Figure 1:**
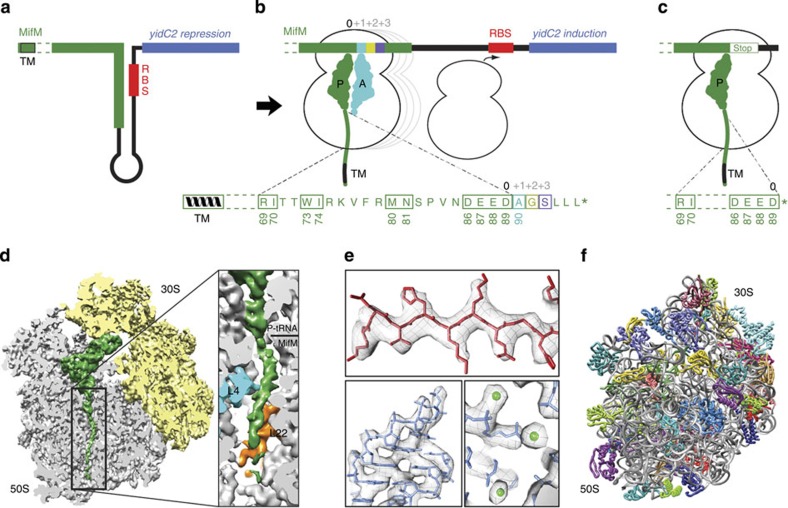
Cryo-EM structure and molecular model of the *B. subtilis* MifM-stalled ribosome complex. (**a**,**b**) Schematic of the *mifM-yidC2* mRNA illustrating the N-terminal transmembrane (TM) segment (black, helix) and C-terminal stalling region (green) of the MifM leader peptide with the stem-loop structure that sequesters the ribosome-binding site (RBS) of the *yidC2* gene (blue). In (**b**) the multisite ribosome stalling (0, +1, +2 and +3) during translation of MifM maintains the unfolded conformation of the mRNA allowing ribosome binding and induction of *yidC2* expression. The MifM stalling sequence (residues 69–89) is shown with critical residues boxed in green. Asterisks indicate stop codons. (**c**) MifM-stalled ribosome complex used for cryo-EM. (**d**) Transverse section of the cryo-EM structure of the MifM-SRC (30S, yellow; 50S, grey) showing P-tRNA and MifM nascent chain (green) within the ribosomal tunnel and enlargement where ribosomal proteins L4 (cyan) and L22 (orange) are coloured. (**e**) Electron density (grey mesh) for selected regions of large subunit ribosomal protein and rRNA of the MifM-SRC. (**f**) Molecular model of the *B. subtilis* 70S ribosome.

To understand the structural basis for the mechanism of MifM-dependent translational stalling and provide structural insight into the species specificity of MifM stalling, we set out to structurally characterize the *B. subtilis* MifM-SRC. Unlike for *E. coli*, translation systems for *B. subtilis* are not commercially available. Therefore, to generate complexes of translating *B. subtilis* ribosomes, we developed and optimized a *B. subtilis* S12 lysate-based *in vitro* coupled transcription–translation system (Supplementary Fig. 1 and Methods). This system was used to prepare *B. subtilis* MifM-SRCs by translation of a template encoding the *B. subtilis* MifM leader peptide (Supplementary Fig. 1 and Methods). Homogeneity of the MifM-SRC was increased by replacing Ala90 of MifM with a UAA stop codon (Supplementary Fig. 1), which arrests ribosomes at the first stall site (Fig. 1c), and thereby prevents further translation and subsequent arrest at downstream stall sites^6^. Like the wildtype MifM-SRC, the MifM-Ala90UAA stalled ribosomes are refractory to the action of puromycin, and are also not subject to the action of the termination release factors RF1 and RF2^6^,^10^. This MifM-SRC (Fig. 1c) was separated from non-translating ribosomes and polysomes using sucrose gradient centrifugation and affinity chromatography, as performed previously for other SRCs^17^,^18^. A single MifM-peptidyl-tRNA product, and no free MifM peptide, was detected in the final MifM-SRC (Supplementary Fig. 1), indicating the homogeneity of the sample and its suitability for structural analysis. A single-particle reconstruction of the MifM-SRC was obtained from 305,045 particles using cryo-EM (Fig. 1d). The MifM-SRC has an average resolution of 3.9 Å, with local resolution calculations^19^ indicating that the resolution of the core of the 50S subunit reaches 3.5 Å (Supplementary Fig. 2). This is consistent with the features of the cryo-EM map, such as strand separation in β-sheets, the pitch of α-helices as well as density for many side chains of the ribosomal proteins (Fig. 1e). In addition, the rRNA backbone and nucleotides are well resolved, as well as the position of many putative magnesium ions (Fig. 1e). The distinct features of the electron density (Supplementary Figs 3 and 4), together with the high homology to *E. coli* and *T. thermophilus* 70S ribosomal structures^20^,^21^, allowed a molecular model of the *B. subtilis* 70S ribosome to be built (Fig. 1f, Table 1 and Supplementary Figs 5 and 6), encompassing a total of 4,579 of the 4,602 (99.5%) nucleotides of the 5S, 16S and 23S rRNAs, 19 small subunit ribosomal proteins and 29 of the 32 large subunit proteins (L1, L7/L12 and L9 were excluded). The slightly lower resolution of the small subunit (4.0 Å, Supplementary Fig. 2) restricted the modelling of the small subunit ribosomal proteins to a backbone trace, whereas sidechains were modelled for the large subunit proteins.

**Figure 2:**
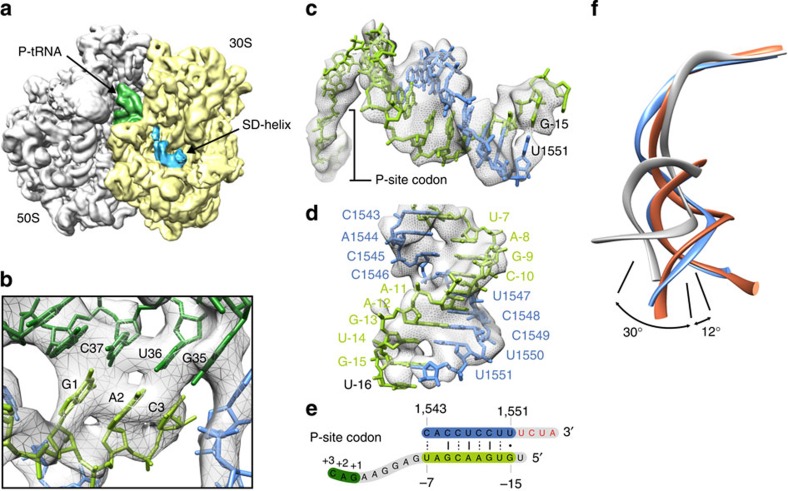
The path of the mRNA through the *B. subtilis* MifM-SRC. (**a**) Location of Shine–Dalgarno(SD)-anti-SD-like helix on 70S ribosome (30S, yellow; 50S, grey, P-tRNA, green). (**b**) Codon–anticodon interaction between P-tRNA (dark green) and mRNA (pale green). (**c**) Electron density (grey mesh) and molecular model for the path of the *mifM* mRNA (green) from the P-site to back of the 30S subunit (**d**) where it forms an 8 base pair SD-anti-SD-like helix with the 3' end of the 16S rRNA (blue). (**e**) Schematic for the non-canonical base-pairing observed in the SD-anti-SD-like helix of the MifM-SRC. (**f**) Comparison of SD-anti-SD-like helix observed in *B. subtilis* MifM-SRC (blue) with initiation SD-anti-SD helix (grey) and post-initiation SD-anti-SD helix (red) observed previously^22^.

The majority of the rRNA in the core of *B. subtilis* 70S ribosome is structurally conserved with *E. coli* and *T. thermophilus* ribosomes, however, specific regions resemble only *E. coli* (h26, h33, h44 in the 30S; H27, H68 and H79 in the 50S) or *T. thermophilus* (h9 in the 30S; H15 and H63 in the 50S), whereas others appear to be *Bacillus* specific (h6, h10, h17 in the 30S; H25 and H54 in the 50S; Supplementary Fig. 8). As expected from the genome sequence, the *B. subtilis* 70S ribosome lacks density for S21 (Supplementary Fig. 9), which is present in *E. coli* but absent in *T. thermophilus*. Surprisingly, density for L9 and L25 (also referred to as the general stress protein CTC) is also absent in the *B. subtilis* 70S ribosome (Supplementary Fig. 9), despite L9 and L25 being encoded in the *B. subtilis* genome. Only one tRNA is present in the MifM-SRC, located at the P-site, where it makes codon–anticodon interactions with P-site codon of the mRNA (Fig. 2a,b). No density for the mRNA is observed in the A-site, nor within the downstream mRNA channel, whereas in contrast, the upstream 5′ end of the mRNA can be unambiguously traced from the P-site through the E-site to the back of 30S subunit where it establishes a number of non-canonical base-pairs with the 3′ end of the 16S rRNA resulting in a 8-basepair helix (Fig. 2c–e), analogous to the Shine–Dalgarno (SD)-anti-SD helices observed during elongation^22^,^23^ (Fig. 2f). The CCA-end of the P-tRNA adopts its canonical position at the PTC of the ribosome, with clear density for the MifM nascent polypeptide chain extending from the ribose of A76 and traversing the entire length of the ribosomal exit tunnel (Fig. 1b). Local resolution calculations indicate that the MifM nascent chain exhibits some degree of flexibility (Supplementary Fig. 2), particularly in regions that do not appear to establish contacts with components of the ribosomal tunnel.

### MifM interactions with the ribosome

**Figure 3:**
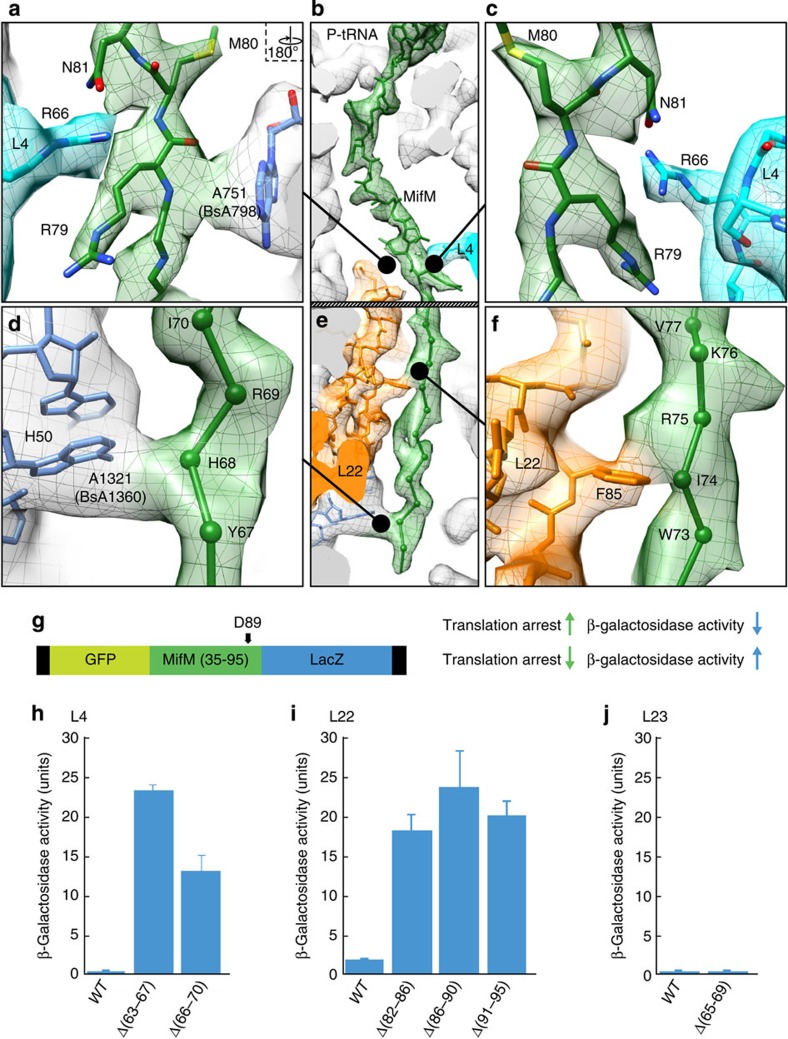
Interactions of the MifM nascent chain with components of the ribosomal tunnel. (**a**–**f**) Contacts between MifM nascent chain (green) and the ribosome in the (**a**–**c**) upper and (**d**–**f**) lower region of the ribosomal tunnel. Electron density (mesh) is coloured for MifM (green), L4 (cyan), L22 (orange) and rRNA (grey). In (**d**–**f**) the cryo-EM map was filtered to 4 Å resolution and the nascent chain modelled as a backbone trace. (**g**) Schematic for the GFP–MifM–LacZ reporter used to monitor translational arrest, where stalling prevents β-galactosidase production in *B. subtilis in vivo*. (**h**–**j**) β-galactosidase activity from the GFP–MifM–LacZ reporter (as in **g**) expressed in *B. subtilis* strains bearing wildtype (WT) or truncated versions of ribosomal proteins (**h**) L4, (**i**) L22 or (**j**) L23. Error bars indicate the s.d. of three independent biological replicates.

In the upper region of the tunnel, the density for the MifM nascent chain is well resolved at the sites of contact, namely adjacent to the PTC and at the constriction, enabling side chains of MifM residues E87/D89 and R79-N81 to be modelled (Fig. 3a–c). In contrast, the MifM nascent chain becomes fragmented in the lower region of the tunnel, indicating a higher degree of flexibility, and was therefore filtered to 4 Å to obtain continuous uninterrupted electron density (Fig. 3d–f). For the less resolved regions, that is, S82-E86 and W73-F78, we present a model for the backbone trace and acknowledge that the register for MifM may become lost after the constriction in the lower regions of the tunnel. The model localizes the three pairs of critical MifM residues, M80-N81, W73-I74 and R69-I70, at the sites of contact with components of the ribosomal tunnel (Fig. 3b,f).

In addition to the PTC (discussed later), four main interactions are observed between MifM and the ribosome: Two contacts are observed at the constriction, which sandwich the MifM nascent chain between Arg66 of L4 on one side of the tunnel and A751 (*E. coli* numbering is used throughout the text) of the 23S rRNA on the other (Fig. 3a–c). The contact to A751 is one of the strongest fusions of density between the tunnel wall and MifM, and appears to involve the backbone in the vicinity of R79 of MifM (Fig. 3a), whereas Arg79 and Asn81 coordinate interaction with the sidechain of Arg66 of L4. Consistently, the N81A mutation that abrogates MifM-stalling^6^,^10^ would also abolish the potential hydrogen bond formed with Arg66 of L4. Two contacts are observed within the lower region of the tunnel, one between Phe85 of L22 and Ile74 of MifM (Fig. 3d) and another between A1321 in H50 of the 23S rRNA in the vicinity of His68 of MifM (Fig. 3f). These contacts are likely to be important since insertions within L22 compromise MifM-dependent stalling, as do mutations of Trp73, Ile74 or His68 to alanine^10^. However, because the H68A only partially affected YidC2 induction using the *mifM-yidC2′-lacZ* reporter^10^, the latter contact with A1321 may instead reflect the importance of the neighbouring MifM residues Arg69 and Ile70 for stalling^10^. While a fully extended backbone conformation for MifM in the lower tunnel region does not bring these residues into direct proximity of A1321, because of the limited resolution for this region of the nascent chain we cannot rule out that the side chain of Arg69 can reach down to directly contact A1321. In agreement with the observed contacts between MifM and the ribosome, we could demonstrate using a *B. subtilis in vivo* GFP–MifM–LacZ (translational fusion) reporter (Fig. 3g) that both L22 and L4, but not L23 (the only other ribosomal protein that contributes to the exit tunnel), are important for MifM stalling (Fig. 3h–j): In a wildtype *B. subtilis* strain, translation of the GFP–MifM–LacZ reporter leads to stalling within the MifM sequence and therefore negligible β-galactosidase is produced (Fig. 3h,j), whereas *B. subtilis* strains bearing internal deletions within the tunnel lumen regions of L22 and L4 lead to an increase in β-galactosidase activity, consistent with a reduction in MifM stalling. In contrast, β-galactosidase activities remained low when deletions were made in L23 (Fig. 3j), consistent with the absence of a direct interaction between MifM and L23 in the MifM-SRC.

### Species specificity of MifM stalling

**Figure 4:**
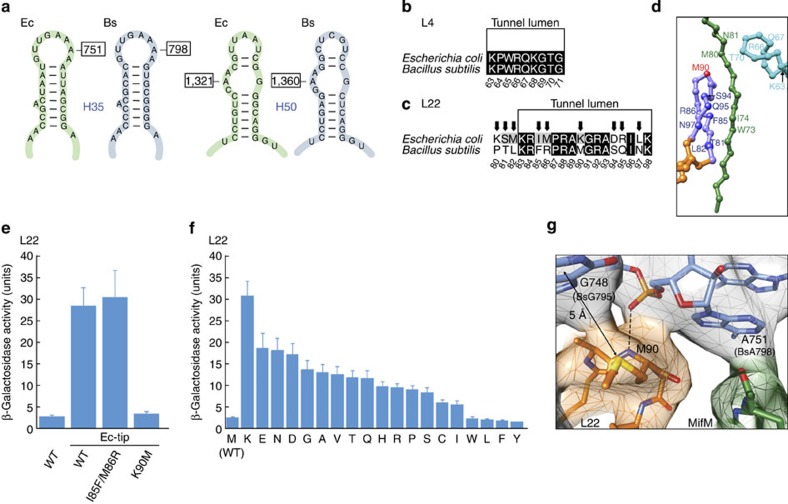
Residue M90 in L22 contributes to the species specificity of MifM stalling. (**a**–**c**) Conservation between *B. subtilis* and *E. coli* of (**a**) 23S rRNA nucleotides A751 (A798 in *B. subtilis*) and A1321 (A1360 in *B. subtilis*) within helices H35 and H50, respectively, as well as the tunnel lumen region of ribosomal proteins (**b**) L4 and (**c**) L22. In (**b**,**c**) similar and identical residues are shaded grey and black, respectively. (**d**) Overview of relative positions of MifM to tunnel lumen residues of L4 and L22. (**e**,**f**) β-galactosidase activity from the GFP–MifM–LacZ reporter (as in Fig. 3g) expressed in *B. subtilis* strains bearing wildtype (WT) L22 compared with (**e**) Ec-tip mutants where *B. subtilis* residues 80–98 are substituted with the equivalent *E. coli* residues (see **d**) and then additionally reverted to *B. subtilis* residues by substitutions I85F/M86R or K90M, or (**f**) L22 mutants where all possible amino-acid substitutions at position M90 of *B. subtilis* L22 were generated. Error bars indicate the s.d. of three independent biological replicates. (**g**) Interaction between the side chain of M90 of L22 with the base of G748 and potential hydrogen bonding (dashed line) between the backbone of M90 and the phosphate-oxygen of A751.

To understand the species specificity of MifM stalling, we analysed the sequence and structure conservation of the ribosomal tunnel between *B. subtilis* and *E. coli*, revealing that the rRNA components are highly conserved, including nucleotides A751 and A1321 (Fig. 4a) and, similarly, the luminal region of L4 is invariant between *B. subtilis* and *E. coli* (Fig. 4b). In contrast, the β-hairpin of L22 exhibits a number of amino-acid differences between *B. subtilis* and *E. coli* (Fig. 4b–d), suggesting that the specificity of MifM stalling may be conferred by L22. To test this possibility, we utilized the GFP–MifM–LacZ reporter assay and demonstrated that replacing the β-hairpin of *B. subtilis* L22 with corresponding sequence from *E. coli* L22 led to an increase in β-galactosidase levels (Fig. 4e), consistent with a reduction in stalling at the MifM-derived sequence. Strikingly, reverting a single amino acid (K90M) in the β-hairpin of L22 from the *E. coli* sequence (K90) back to *B. subtilis* (M90) was sufficient to restore the low levels of β-galactosidase activity indicative of efficient stalling at MifM, whereas other reversions, such as the IM to FR double mutation at positions 85–86 of L22 retained the elevated β-galactosidase levels (Fig. 4e). These findings suggest that the nature of the amino acid at position 90 of L22 can markedly influence the efficiency and specificity of stalling. Therefore, we introduced all 19 amino-acid substitutions at position 90 of *B. subtilis* L22 and monitored the β-galactosidase activity using the GFP–MifM–LacZ reporter (Fig. 4f). A wide range of β-galactosidase activities was observed, with K90, as present in *E. coli* L22, producing the highest levels, whereas hydrophobic or aromatic amino acids, such as F, Y, L and I, in position 90 exhibited β-galactosidase activities similar to the wildtype L22 with M90 (Fig. 4f). While γ-proteobacteria, such as *E. coli* usually have K90 and most *Bacillus* species have M90, other bacterial lineages generally contain positively charged amino acids such as R, Q, K or H at the equivalent position (Supplementary Fig. 10), and are therefore unlikely to promote efficient MifM stalling.

M90 of L22 does not directly contact MifM but rather forms a Met–Sulfur aromatic interaction with G748 of the 23S rRNA (Fig. 4g), suggesting that the influence of this residue on MifM stalling may be indirect. One possibility is that M90 influences the interaction of MifM with nucleotide A751, either indirectly through G748, or more directly since the backbone nitrogen of M90 is within hydrogen bonding distance of the phosphate-oxygen of A751 (Fig. 4g). In fact, the entire tip of the β-hairpin of L22 forms an intricate network of interactions with the 750-loop of 23S rRNA helix H35 (Supplementary Fig. 11), suggesting that M90 mutations which alter the interaction with G748 as well as the conformation of the L22 tip could lead to rearrangements in the A750-loop. While the A751 region is structurally similar between *B. subtilis* and *E. coli*, the resolution does not allow us to exclude subtle changes or shifts in the positions of the nucleotides that could influence interaction with the MifM nascent chain. In addition, M90 mutations could alter the conformation of the β-hairpin of L22 and thereby influence the interaction between MifM and F85 of L22 (Fig. 3f). Support for the interplay between the β-hairpin of L22 and MifM residues located below the constriction comes from rescue experiments: Random mutagenesis of the β-hairpin (positions 80–89 and 91–98) of *B. subtilis* L22-M90K was performed to search for second site mutations that restore MifM stalling and induce expression of a YidC2′–LacZ fusion, which led to the identification of mutations at position G91 of L22 that restored induction by up to ∼70% of the wildtype level, as well as lesser effects at positions R92, A93 and F85 (Supplementary Fig. 12). Second site mutations that could restore MifM stalling and induce expression of a YidC2′–LacZ fusion in the presence of *B. subtilis* L22-M90K were also identified within the MifM nascent chain. In this latter case, random mutagenesis was performed at MifM positions 72–81, leading to the identification of the T72R mutation that restored induction by up to ∼60% of the wildtype level, with lesser effects also being observed at positions R75, K76 and F78 (Supplementary Fig. 12).

### MifM inactivation of the PTC

**Figure 5:**
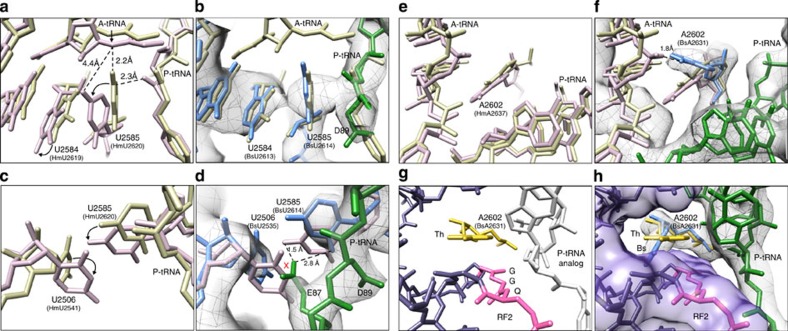
MifM stabilizes the uninduced state of the PTC to inhibit A-tRNA accommodation. (**a**) A-tRNA accommodation leads to conformational changes in U2584 and U2585 shifting the PTC from an uninduced (yellow) to an induced state (salmon)^24^,^25^,^26^. (**b**) The PTC of the MifM-SRC (grey density with rRNA (blue) and P-tRNA (green)) resembles that of the uninduced state (yellow). (**c**) Rotation of U2506 is required to accommodate the shift in U2585 that occurs on A-tRNA accommodation^24^,^25^,^26^. (**d**) E87 of MifM nascent chain (green) occupies the position of the induced state of U2506. (**e**) A-tRNA accommodation leads to a slight shift in the position of A2602. (**f**) The position of A2602 (blue) in the MifM-SRC encroaches on the A-tRNA binding site and is distinct from the position of A2602 in the uninduced (yellow) or induced states (pink)^24^,^25^,^26^. (**g**) A distinct conformation of A2602 (yellow) is required to position the GGQ motif of RF2 at the PTC to catalyse peptidyl-tRNA hydrolysis^29^,^30^,^31^,^32^. (**h**) The position of A2602 (blue) in the MifM-SRC is incompatible with the canonical binding position of RF2 at the PTC^29^,^30^,^31^,^32^.

To understand how the interaction of the MifM nascent chain with components of exit tunnel prevents stable binding of the A-tRNA and therefore leads to inhibition of peptide bond formation, we compared the conformation of nucleotides at the PTC in different states of peptide bond formation^24^,^25^,^26^,^27^ with the PTC of the MifM-SRC (Fig. 5a–c). U2584 and U2585 shift by 2–3 Å on A-tRNA accommodation, that is, when moving from the unaccommodated (uninduced) to the accommodated (induced) state^24^,^25^,^26^ (Fig. 5a). In the MifM-SRC, U2584 and U2585 resemble the uninduced state, consistent with the absence of A-tRNA (Fig. 5b). The shift in U2585 that occurs during A-tRNA accommodation also requires a corresponding rotation of U2506 (Fig. 5c). In the MifM-SRC, the side chain of E87 overlaps in position with the induced conformation of U2506 (Fig. 5d), suggesting that the MifM nascent chain prevents A-tRNA accommodation by sterically preventing PTC nucleotides U2506, and thereby also U2585, from adopting their induced conformational states. E87 comprises part of the DEED sequence that is critical for MifM stalling^2^, however, single mutation of any of the amino acids within the DEED motif, including E87A, does not markedly reduce stalling^6^. To completely abolish stalling, multiple D/E to A mutations within this motif are required^6^, indicating some functional redundancy. One could speculate that within the context of the E87A mutation the role of E87 in blocking the U2506 shift is assumed by a neighbouring D/E amino acids, however, without determining structures of the individual mutant MifM-RNCs it would be difficult to address this question.

In addition, during normal translation A2602 undergoes a slight shift on A-tRNA accommodation (Fig. 5e), whereas in the MifM-SRC, A2602 appears to adopt a defined position (Fig. 5f), which would restrict the entry of the incoming aminoacyl-tRNA to the A-site of the PTC. The conformation of the PTC of the MifM-SRC also provides an explanation as to why the MifM-stalled ribosomes are resistant to the action of the RF1 and RF2 when the A90 position is a stop codon^6^,^10^: Peptidyl-tRNA hydrolysis by RF1 and RF2 requires the accurate placement of the GGQ motif in domain 3 at the PTC of the ribosome, which induces specific conformations of PTC nucleotides, such as A2602^28^,^29^,^30^,^31^,^32^ (Fig. 5g). This conformational change would be incompatible with the uninduced state of the PTC observed in the MifM-SRC (Fig. 5h). Indeed, the conformation of A2602 in the MifM-SRC is similar to that observed in the cryo-EM structure of the TnaC-SRC^33^, where translational arrest occurs because RF2-dependent peptidyl-tRNA hydrolysis is inhibited^7^.

Although our structure does not provide direct insight into the mechanism of PTC inhibition when the ribosome stalls at subsequent downstream residues, we note that the SD-like helix formed between the MifM mRNA and the 5′ end of the 16S rRNA (Fig. 2) is likely to contribute to multisite stalling, as SD-like sequences within the open reading frames of mRNAs have been reported to induce translational pausing in bacteria^34^. However, the introduction of frameshifts within the MifM leader peptide abolished translational stalling at all sites^10^, indicating that the SD-like helix alone is insufficient to induce pausing independently of the MifM nascent chain-induced stall, possibly because the helix cannot form in the absence of stalling or that SD-dependent pausing is actually negligible^35^.

## Discussion

**Figure 6:**
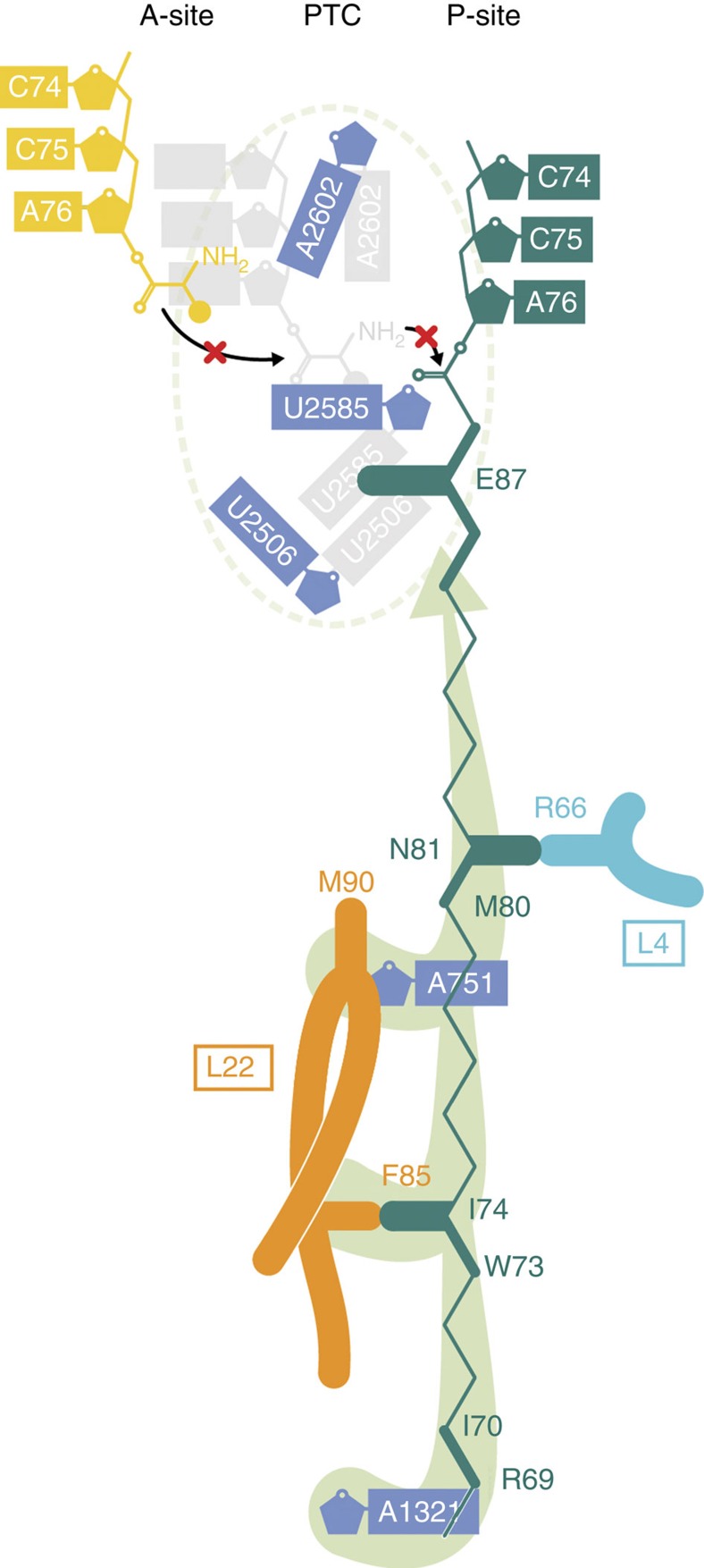
Molecular basis for the specificity and mechanism of MifM-dependent translation arrest. Model illustrating how interactions of MifM within the ribosomal tunnel stabilize a specific conformation of E87 that prevents conversion of the uninduced state to the induced state and thereby blocks aminoacyl-tRNA binding at the A-site of the PTC.

Collectively, our biochemical and structural findings lead us to propose a model for the mechanism and specificity of MifM stalling (Fig. 6): Residue M90 of L22 contributes to the species specificity of stalling, either through structural constraint given to A751 or residue F85 of L22. In the absence of any obvious relay of conformational changes to the PTC through the rRNA, we propose that these interactions, together with contacts between MifM and A1321 in 23S rRNA helix H50 and R66 of L4, promote a defined conformation of the MifM nascent chain such that residue E87 of MifM interacts with U2506 and U2585, thereby stabilizing the uninduced state of the PTC and preventing accommodation of the incoming A-tRNA (Fig. 6). In addition, we envisage that the pulling force exerted on the MifM nascent chain by interaction of the MifM TM with the YidC1/2 translocon during membrane insertion would release the MifM-dependent stalling by disrupting interactions between MifM and the ribosomal tunnel as well as displacing the E87 side chain of MifM and thus allowing U2506 and U2585 to adopt the induced state of the PTC that is necessary for aminoacyl-tRNA accommodation at the A-site. The mechanism of ligand-independent translation arrest by MifM as described here is structurally distinct from previously characterized peptide-dependent stalling systems, such as Erm^36^,^37^, TnaC^33^,^38^ and SecM^18^, illustrating the plasticity of the ribosome to employ diverse mechanisms to sense the nascent chain in the tunnel and evoke silencing of the PTC.

## Methods

### Strain and plasmid construction

*B. subtilis* strains and plasmids used in this study are listed in Supplementary Tables 1 and 2, respectively. *B. subtilis* strains were derivatives of PY79 (wildtype)^39^ and constructed by transformation with either plasmid or purified *B. subtilis* chromosomal DNA. Plasmids were constructed by standard cloning methods, fusion PCR and site-directed mutagenesis^40^ using primers and template plasmids listed in Supplementary Tables 2 and 3. Constructions of plasmids pCH735, pCH835, pCH913, pCH1142 and pEB71 were described previously (references shown in Supplementary Table 2). Plasmid pCH1517 was constructed by cloning an SphI-BglII-digested PCR fragment amplified from pCH913 using primers SP26 and SP27 into the SphI-BamHI site of pCH735. Plasmid pCH1570 was constructed by cloning a BamHI-SphI fragment of pCH1567 into pCH1142. Plasmid pCH1567 was constructed by cloning a XbaI-SphI-digested PCR fragment amplified from PY79 chromosomal DNA using primers SP66 and SP67 into pCH1557. Plasmid pCH1557 was constructed as follows. A PCR fragment amplified from PY79 chromosomal DNA using primers SP68 and SP69 and another PCR fragment amplified from pEB71 using primers SP70 and SP71 were fused by the following PCR using primers SP68 and SP72, digested with both BamHI and XbaI and then cloned into pUC118. Plasmid pCH1587 was constructed as follows. A PCR fragment amplified from PY79 chromosomal DNA using primers SP28 and SP29 and another PCR fragment amplified from pEB71 using primers SP30 and SP31 were fused by the following PCR using primers SP28 and SP31, digested with both SacI and SphI and then cloned into pCH1142. Plasmid pCH1901 was constructed as follows. A PCR fragment amplified from pCH1587 using primers SP34 and SP35 and another PCR fragment amplified from pCH1587 using primers SP36 and SP37 were fused by the following PCR using primers SP34 and SP37, digested with both XhoI and SphI and then cloned into pCH1590. Other plasmids were constructed by site-directed mutagenesis using primers and templates shown in Supplementary Tables 2 and 3.

### *Bacillus subtilis* S12 translation extract

The *B. subtilis* S12 translation extract was prepared following a procedure described for *E. coli* S12 translation extract^41^,^42^, with some modifications. Briefly, cells (*B. subtilis* strain 168) were grown to OD_600_ 4.5 in an ‘INFORCE HT minifors' bench-top fermenter in 2 × YPTG medium (16 g l^−1^ peptone, 10 g l^−1^ yeast extract, 5 g l^−1^ NaCl, 22 mM NaH_2_PO_4_, 40 mM Na_2_HPO_4_, 19.8 g l^−1^ glucose (sterile filtered)), with extra glucose feeding (feed: 10) at 37 °C while maintaining pH 7.0 and oxygen level (60%). Cells were collected at 5,000 × *g* at 4 °C for 15 min and subsequently washed 3 × in cold Buffer A (10 mM Tris–acetate (pH 8.2), 14 mM magnesium acetate, 60 mM potassium glutamate, 1 mM dithiothreitol and 6 mM 2-mercaptoethanol). Cells were snap-frozen in liquid nitrogen and stored at −80 °C. About 15 g of cells were thawed on ice, resuspended in 10 ml of cold buffer B (buffer A without 2-mercaptoethanol) and broken open in an ‘microfluidics model 110I lab homogenizer', 3 × at 15,000 psi. The lysate was cleared subsequently at 12,000 × *g* and 4 °C for 10 min and incubated for 30 min at 37 °C in a water bath. The cell extract was aliquoted, snap frozen and stored at −80 °C.

### PCR and *in vitro* transcription

PCR reaction (Phusion Master Mix (NEB), 98 °C 5 min (98 °C 5 s; 52 °C 5 s; 72 °C 20 s) 28 × 72 °C 10 s, 12 °C) was prepared with MifMfor and MifMrev (Supplementary Table 3) and template DNA containing the sequence: (5′-TAATACGACTCACTATAGGGCGAATTGGCGGAAGGCCGTCAAGGCCACGTGTCTTGTCCATTAATTAACGTTTAACTTTAAGAAGGAGATATACCAATGGGTCATCACCATCACCATCACCATCACGATTACGATATTCCAACGACCCTGGAAGTTCTGTTCCAGGGACCCGGTACCATGTTTGTGGAATCGATAAATGACGTTTTATTCTTAGTCGATTTTTTCACAATTATTCTTCCTGCTCTAACGGCAATCGGGATTGCATTCCTCTTACGGGAGTGCCGTGCGGGCGAGCAATGGAAATCAAAACGAACAGATGGGCCCTACCCATACGATGTTCCAGATTACGCTGACTTTCTTATTATTATATATCATCGCATTACAACTTGGATACGTAAAGTCTTCCGCATGAATTCGCCTGTGAACGATGAGGAAGACGCCGGTTCTCTTCTTTTATAA-3′; underlined are the T7 promoter region, ribosomal binding site, start codon, 8 × His-tag and stop codon, respectively). PCR product was purified via spin columns and *in vitro* transcription reaction was set up using 1 μg PCR product per 50 μl reaction volume and T7 RNA polymerase. RNA was purified by LiCl/ethanol precipitation.

### Preparation of the MifM-SRC

A volume of 1,360 μl of reaction mix containing 240 mM HEPES-KOH (pH 8.2), 60 mM glucose, 1.2 mM ATP, 0.85 mM CTP, GTP, and UTP, 2 mM DTT, 0.17 mg ml^−1^ *E. coli* total tRNA mixture (from strain MRE600), 90 mM potassium glutamate, 80 mM of ammonium acetate, 8 mM magnesium acetate, 20 mM potassium phosphate dibasic (pH 7.2), 34 μg ml^−1^ L-5-formyl-5, 6, 7, 8-tetrahydrofolic acid (folinic acid), 2.1 mM (each) amino-acid mix, 2 mM cysteine, 2% (w/v) PEG 8000 was preheated to 30 °C before the addition of 540 μl (27% (v/v)) cell extract (S12 extract). The whole reaction was incubated at 30 °C for 2 min before the addition of 100 μl *mifM* mRNA (Supplementary Fig. 1) and further incubated at 30 °C for 33 min, shaking at 1,000 r.p.m.

### Purification of the MifM-SRC

*In vitro* translation reactions (4 × 500 μl) were loaded onto 500 μl sucrose cushion (750 mM sucrose) in Buffer C (50 mM HEPES, 250 mM KOAc, 10 mM MgOAc, 0.1% DDM, 1/1,000 complete protease inhibitor (Roche), 0.2 U ml^−1^ RNasin, pH 7.2 at 4 °C) and centrifuged for 150 min (45,000 r.p.m., 4 °C) in a Beckman Coulter TLA 120.2 fixed-angle rotor. Pellets were resuspended in 4 × 300 μl 250 mM sucrose in ice cold buffer C and loaded onto a Talon metal affinity chromatography column (750 μl resin) pre-equilibrated in 10 ml buffer C containing 10 μg μl^−1^ bulk tRNA. The column was washed with 25 ml buffer C until no significant absorption (A_260_) could be detected in the wash fractions. The MifM-SRC, bound to the Talon matrix by MifM's N-terminal 8 × His-tag, was eluted in 4 × 500 μl buffer C containing 150 mM Imidazole. The eluate was loaded onto 10–40% sucrose gradients (prepared with buffer C) and centrifuged for 13 h in a Beckman coulter SW40 Ti swinging bucket rotor (20,000 r.p.m., 4 °C). Gradients were separated on a Biocomp Gradient Station *ip* and fractions containing 70S ribosomal particles were collected and pelleted for 3 h in a Beckman Coulter TLA 120.2 fixed-angle rotor (45,000 r.p.m., 4 °C). The MifM-SRC pellet was resuspended in 28 μl 30 mM sucrose in buffer C, on ice (69 OD ml^−1^), aliquoted and snap-frozen. Samples were further analysed by SDS–PAGE and western blotting.

### MifM-SRC/SRP sample and cryogrid preparation

For grid preparation 2.5 μl *in vitro* reconstituted *B. subtilis* signal recognition particle (20 pmol μl^−1^ in SRP buffer: 25 mM HEPES, 100 mM KOAc, 10 mM MgOAc, 1 mM DTT, 10% Glycerol, pH 7.2 at 4 °C) were diluted to a final volume of 44.2 μl with buffer E (50 mM HEPES, 250 mM KOAc, 2 mM 2-mercaptoethanol, 0.06% Nikkol, pH 7.2 at 4 °C) and activated by incubation at 30 °C for 10 min. Subsequently, 5.8 μl (9.6 pmol) of MifM-SRC was added and incubated for 10 min at 30 °C. Finally the prepared mix was diluted with 22.5 μl buffer F (50 mM HEPES, 250 mM KOAc, 2 mM 2-mercaptoethanol, pH 7.2 at 4 °C) for optimal grid coverage.

### Cryoelectron microscopy and single particle reconstruction

The MifM-SRC/SRP sample was applied to 2 nm precoated Quantifoil R3/3 holey carbon supported grids and vitrified using a Vitrobot Mark IV (FEI Company). Data collection was performed using the EPU software (FEI) at NeCEN (Leiden, Netherlands) on a Titan Krios transmission electron microscope (FEI, Eindhoven, Netherlands) equipped with a Falcon II direct electron detector. Data were collected with the microscope set to 300 kV, a magnification of 125,085x (pixel size: 1.108 Å) in a defocus range of 1.0–2.5 μm (Table 1). The data were provided as a series of seven frames (grid was pre-exposed for 55 ms, 4 e^−^ Å^−2^ before recording; dose per recorded frame: 8 e^−^ Å^−2^, exposure-time/ recorded frame: 110 ms). Frames 1–3 (accumulated dose of 28 e^−^ Å^−2^) were summed after alignment using Motion Correction software^43^. Images were processed using a frequency-limited refinement protocol that helps prevent over-fitting^44^, specifically by truncation of high frequencies (in this case at 8 Å). As reported and expected^44^, we found that using this processing regime the 0.143 Fourier Shell Correlation (FSC) value provides a good indicator for the true average resolution of the map (Supplementary Fig. 2). In addition, the local resolution of the final map was calculated using ResMap^19^. Power spectra and defocus values were determined using the SPIDER TF ED command and recorded images were manually inspected for good areas and power-spectra quality. Data were processed further using the SPIDER software package^45^, in combination with an automated workflow as described previously^46^. After initial, automated particle selection based on the program SIGNATURE^47^, initial alignment was performed with 529,488 particles, using an *E. coli* 70S ribosome as reference structure^36^. After removal of noisy particles (101,109 particles; 19%), the data set was further sorted using an incremental K-means-like method of unsupervised 3D sorting^48^. Particles lacking density for P-tRNA (75,984 particles; 14%) and those that did not improve in resolution during refinement were omitted from the data set. The major subpopulation (305,045 particles; 58%) showed the presence of stoichiometric densities for P-tRNA and could be refined to an average resolution of 3.9 Å (0.143 FSC) and a local resolution extending to 3.5 Å for the core of the ribosome as computed using ResMap^19^ (Supplementary Fig. 2) and was used for modelling. Subsequently, this main volume could be sorted further into a subvolume containing the signal recognition particle bound to the ribosome (158,726 particles; 30%), which will be described elsewhere (Beckert *et al*., unpublished). The final volumes were subjected to the program EM-BFACTOR^49^ in order to apply an automatically determined negative B-factor for sharpening of the map.

### Molecular modelling and map-docking procedures

The *B. subtilis* 5S, 16S and 23S rRNA sequences were taken from GeneBank; Gene ID 2914271, 936774 and 939981, respectively. Structure-based sequence alignments were generated using Sequence to Structure (S2S)^50^ based on X-ray structure derived models of the small and large ribosomal subunit of *E. coli* (PDB ID 4KIX/Y)^51^. Molecular dynamics flexible fitting (MDFF)^52^ in VMD^53^ was used for initial fitting and refinement of the rRNA models into the electron density. The resulting models of the *B. subtilis* rRNAs were manually inspected and adjusted according to features of the electron-density using Coot^54^, followed by refinement using erraser^55^ and the real-space refine tool in PHENIX^56^ (Table 1). A total of 29 of the 32 large subunit proteins (L1, L7/L12 and L9 were excluded) from the *B. subtilis* 50S subunit were generated using the homology with the equivalent *E. coli* and *T. thermophilus* protein templates (PDB codes 4KIX and 3I8I, respectively). The models were initially fitted to the density using Chimera^57^, followed by real-space refinement using PHENIX^56^ (Table 1). For the 19 proteins of the *B. subtilis* 30S subunit, homology models were generated based on protein templates from *T. thermophilus* (PDB code 3V2C) using HHPred^58^ and fitted to the density using Chimera^57^ and refined in Coot^54^. The resolution of the core of the large subunit (3.5 Å, Supplementary Fig. 2) enabled the majority of the sidechains for the large ribosomal proteins to be modelled (Supplementary Fig. 3), whereas the lower resolution of the core of the small subunit (4.0 Å, Supplementary Fig. 2) limited the molecular modelling to the backbone of the small subunit ribosomal proteins. The fit of the atomic models was validated as described^59^ by calculating the FSC between a theoretical density derived from the atomic models for the *B. subtilis* 70S ribosome (generated using the CP FROM PDB SPIDER command) and the unsharpened cryo-EM map of MifM-SRC (Supplementary Fig. 4). The volumes were multiplied (using the MU SPIDER command) with a ‘soft' mask generated from the theoretical model density (using the TH M and FQ NP SPIDER commands) previous to calculating their Fourier shell correlation (using the RF 3 SPIDER command).

### Figure preparation

Figures showing electron densities and atomic models were generated using UCSF Chimera^57^.

### β-galactosidase assays

*B. subtilis* cells were cultured at 37 °C in LB media for β-galactosidase activity assay^60^. Cells were collected from cultures of OD_600_=0.5–1.0. To test β-galactosidase activities on agar plates, cells were grown on Difco sporulation medium (DSM) agar plates containing 60 μg ml^−1^ 5-bromo-4-chloro-3-indolyl-β-D-galactoside (X-gal) at 37 °C^6^.

### Isolation of suppressor mutants

Plasmid library for isolation of intragenic suppressor mutants of the *rplV(M90K)* mutant were prepared as follows. The second point mutation was introduced into either of the 80–89th or the 91st–98th codons of *rplV(M90K)* on the original plasmid pCH1897 by site-directed mutagenesis using random mixed mutagenic primers listed in Supplementary Table 3 (SP38-SP55). *B. subtilis* strain SCB3378 (*gfp-mifM*^*35*−*95*^*-yidC2′-lacZΩcat, rplV94*) was transformed with the mutagenized plasmid library and then blue colonies were isolated on DSM agar plates including 3 μg ml^−1^ kanamycin and 60 μg ml^−1^ X-gal at 37 °C. The original strain SCB3378 harbours the *rplV94* mutation, which has a seven codon insertion within the *rplV*, resulting in arrest-defective phenotype. Strain SCB3378 was made from SCB824 (*gfp-mifM*^*35*−*95*^*-yidC2′-lacZΩcat, rplV*^*+*^) by isolating a spontaneous erythromycin resistant mutant. Suppressor *mifM* mutants were isolated as follows: Plasmid library was prepared by site-directed mutagenesis using template plasmid pCH835 (*gfp-mifM*^*35*−*95*^*-yidC2′-lacZΩcat*) and random mixed mutagenic primers listed in Supplementary Table 3 (SP56–65) that are designed to introduce a point mutation into either one of the 72nd–81st codons of *mifM*. *B. subtilis* strain SCB3348 (*rplV(M90K)*) was transformed with the mutagenized plasmid library and then blue colonies were isolated on DSM-chloramphenicol-X-gal agar plates at 37 °C.

## Author contributions

D.S. prepared translation extract, MifM-SRC complex performed single-particle cryo-EM analysis and model building. S.C. and N.S.-C. performed *in vivo Bacillus studies*. D.S. and A.I. performed the model refinement and validation. O.B. helped with sample preparation. All authors interpreted the data and helped with the manuscript preparation. D.S. and D.N.W. wrote the manuscript.

## Additional information

**Accession codes.** The cryo-EM map and associated atomic coordinates have been deposited in the EMDB and PDB with the accession numbers EMD-6306 and PDB ID 3J9W, respectively.

**How to cite this article:** Sohmen, D. *et al*. Structure of the *Bacillus subtilis* 70S ribosome reveals the basis for species-specific stalling. *Nat. Commun*. 6:6941 doi: 10.1038/ncomms7941 (2015).

## Supplementary Material

Supplementary Information Supplementary Figures 1-12, Supplementary Tables 1-3 and Supplementary References

## Figures and Tables

**Table 1:** Refinement and Model Statistics.

*Data collection and refinement*	
Particles	305,045
Pixel size (Å)	1.108
Defocus range (μm)	1.0–2.5
Voltage (kV)	300
Electron dose (e^−^/Å^−2^)	28
Map sharpening B factor (Å^2^)	−124.35
Resolution (Å, 0.143 FSC)	3.9
	
*Model Composition*	
Non-hydrogen atoms	135,413
Protein residues	5660
RNA bases	4675
	
*R.m.s. deviations*	
Bonds (Å)	0.0094
Angles (Å)	1.46
	
*Validation (proteins)*	
Molprobity score (79th percentile)	1.94
Clashscore, all atoms (80th percentile)	8.38
Good rotamers (%)	99.85
	
*Ramachandran plot*	
Favored (%)	91.95
Outliers (%)	1.98
	
*Validation (RNA)*	
Correct sugar puckers (%)	97.75
Good backbone conformation (%)	100

FSC,Fourier Shell Correlation; R.m.s., root-mean square.
