# Supplementary material for: Structure of the Bacillus subtilis 70S ribosome reveals the basis for species-specific stalling
Source: Nat Commun. 2015 Apr 23;6:6941. doi: 10.1038/ncomms7941 (PMC4423224; doi:10.1038/ncomms7941)
Supplement: Supplementary Information — Supplementary Figures 1-12, Supplementary Tables 1-3 and Supplementary References [file ncomms7941-s1.pdf]

Supplementary Figures

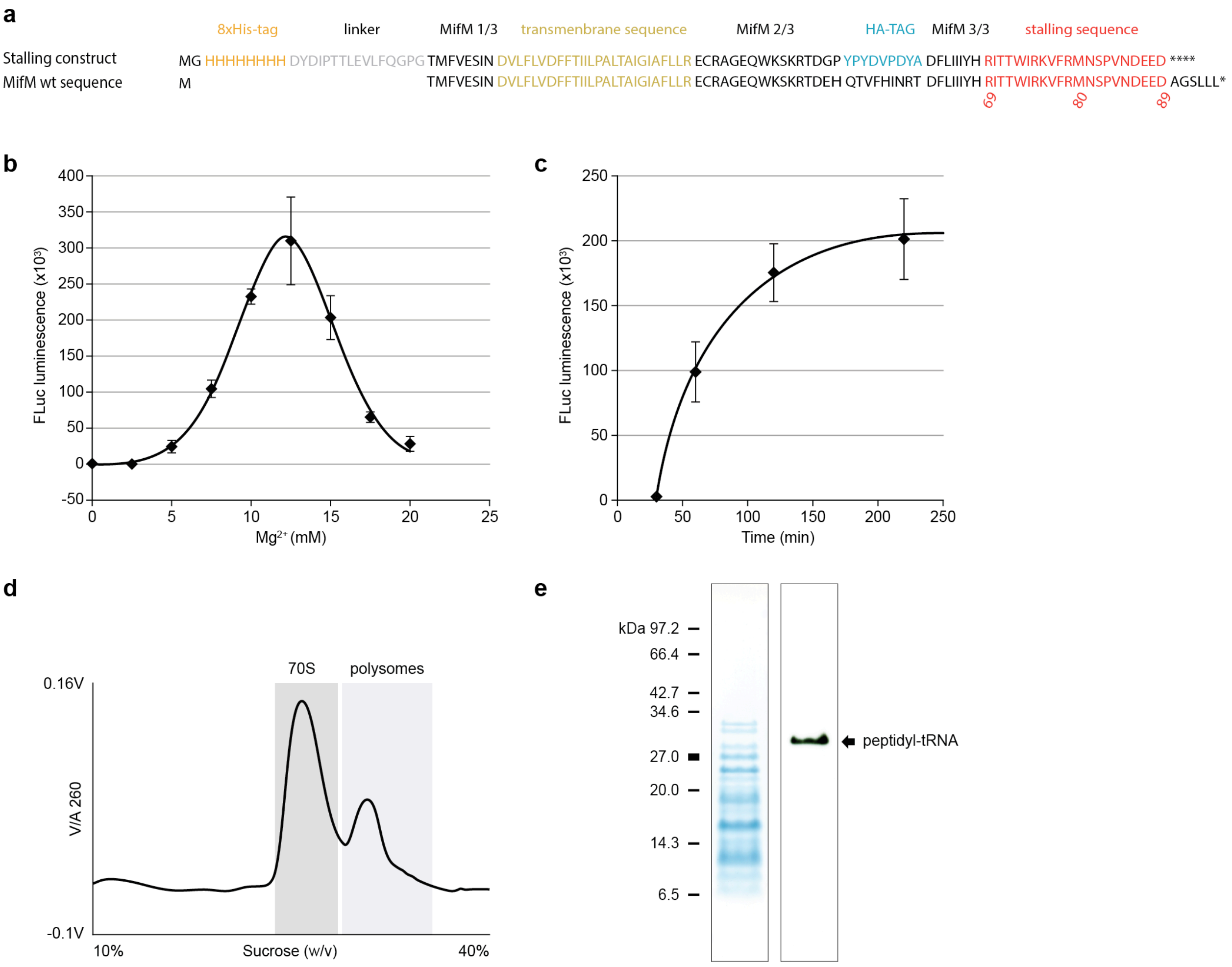

Supplementary Figure 1: Preparation of the MifM-SRC.

a, Comparison of the amino acid sequence of wildtype MifM with the stalling construct used to generate the MifM-SRC. The MifM stalling window (red), transmembrane segment (gold), 8xHis-tag for purification (orange) and HA-tag (cyan) for detection by Western blotting are coloured. **b**,**c**, Optimization of **b**, magnesium (Mg^{2+}) concentration and **c**, time (min) for translation of firefly luciferase (Fluc) in the *B. subtilis* S12 lysate-based *in vitro* translation system. Fluc activity was monitored using luminescence and the error bars represent the standard deviation from the mean for triplicate reactions. **d**, Sucrose gradient profile of translation reaction of MifM stalling construct (from a) used in the optimized *B. subtilis* S12 lysate *in vitro* translation system. Fractions from the 70S peak were collected to remove polysomes. **e**, SDS-PAGE and HA-tag Western blotting revealed a single band for the MifM peptidyl-tRNA and no free MifM peptide.

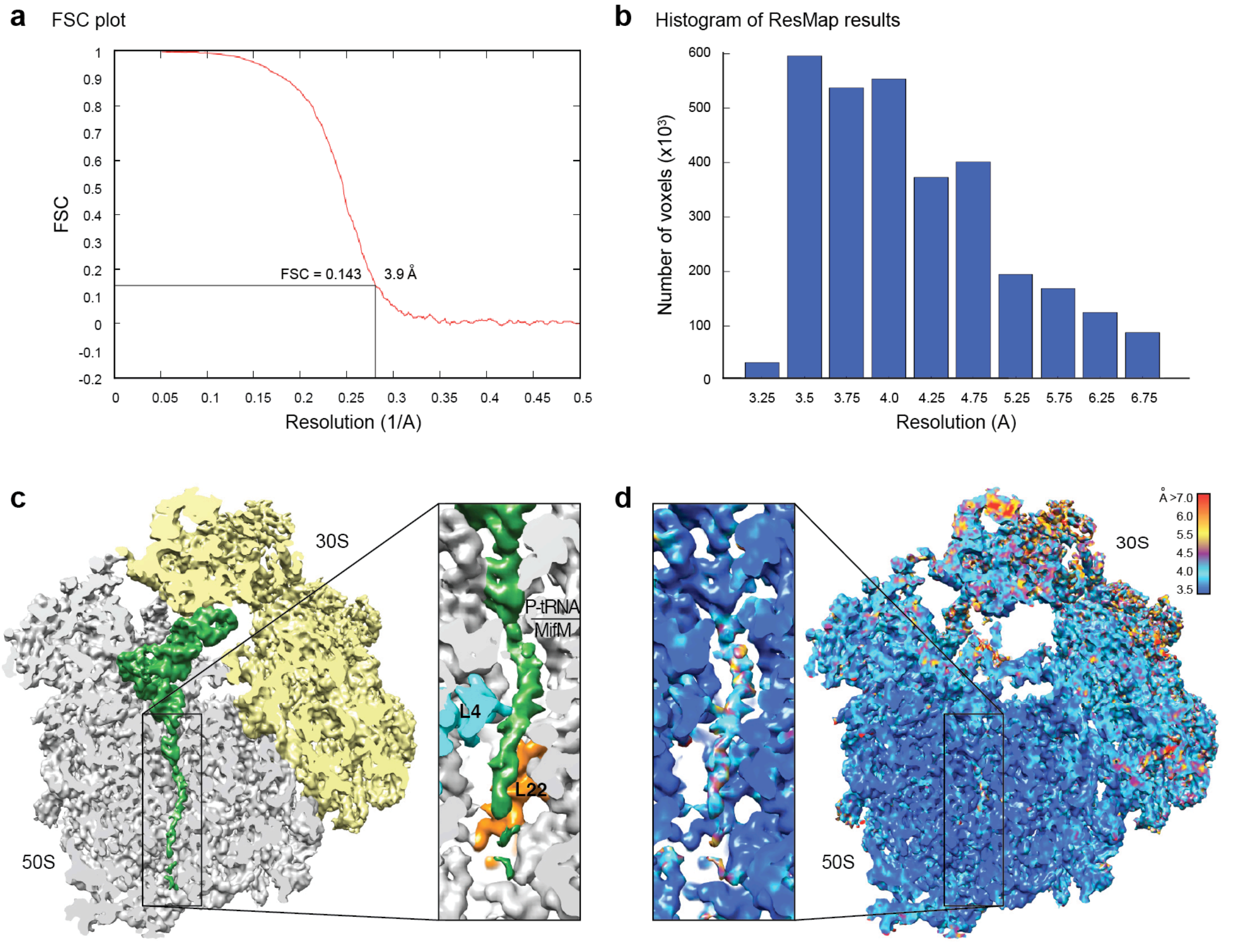

Supplementary Figure 2: Average and local resolution of the cryo-EM map of the MifM-SRC

a, Average resolution of the MifM-SRC was 3.9 Å using the Fourier shell correlation (FSC) cut-off value of 0.143, which was employed since the microscopy images were processed in the absence of spatial frequencies higher than 8 Å[1]. **b**, Histogram generated by ResMap[2] showing that the local resolution of the final MifM-SRC map reaches resolutions of 3.5 Å. **c**, Transverse section of the cryo-EM structure of the MifM-SRC (30S, yellow; 50S, grey) showing P-tRNA and MifM nascent chain (green) within the ribosomal tunnel and enlargement where ribosomal proteins L4 (cyan) and L22 (orange) are coloured. **d**, Same views as **c** but coloured according to local resolution as determined using ResMap[2].

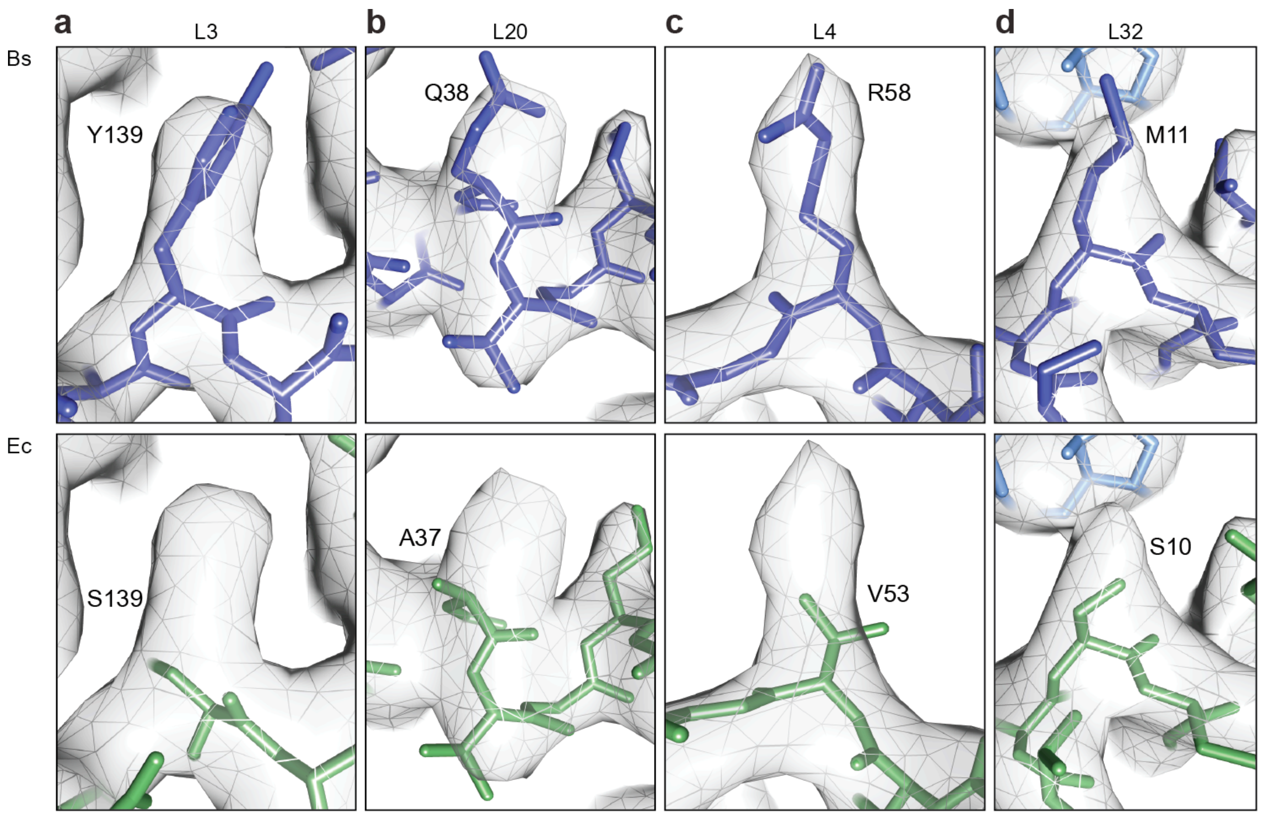

Supplementary Figure 3: Differences in amino acid sequence for ribosomal proteins of the *B. subtilis*, *E. coli* and *T. thermophilus* 50S subunit

a-d, Comparison of selected regions where single amino acid positions differ between *B. subtilis* (blue) and *E. coli* (green, PDB4KIX/Y)[3, 4]. The electron density for the MifM-SRC is shown as a grey mesh.

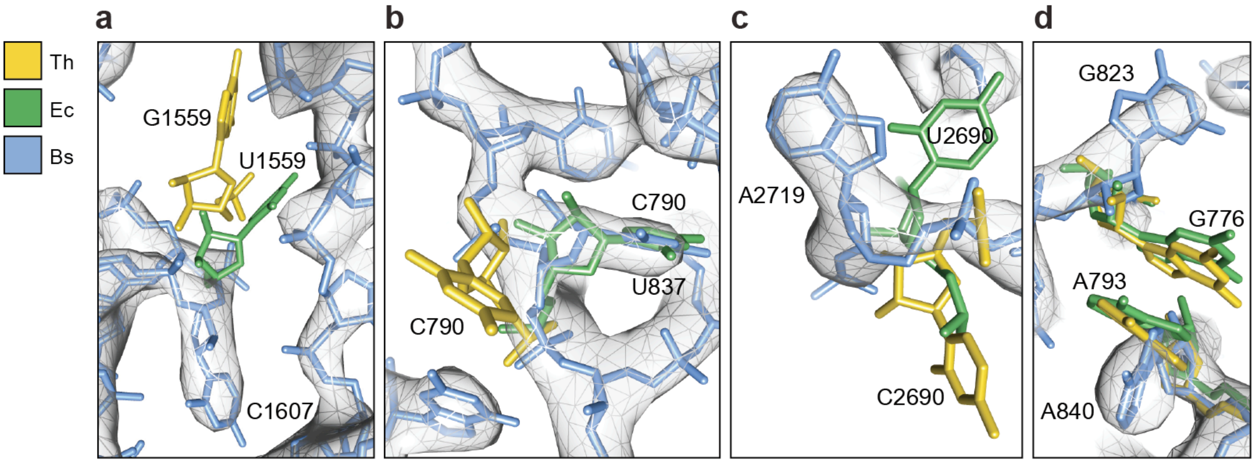

Supplementary Figure 4: Differences in conformation of rRNA nucleotides between *B. subtilis*, *E. coli* and *T. thermophilus* ribosomes

a-d, Comparison of selected regions where single rRNA nucleotide positions differ between *B. subtilis* (blue), *E. coli* (green, PDB4KIX/Y)[3, 4] and *T. thermophilus* (yellow, PDB3I8H/I)[5] 70S ribosomes. The electron density for the MifM-SRC is shown as a grey mesh.

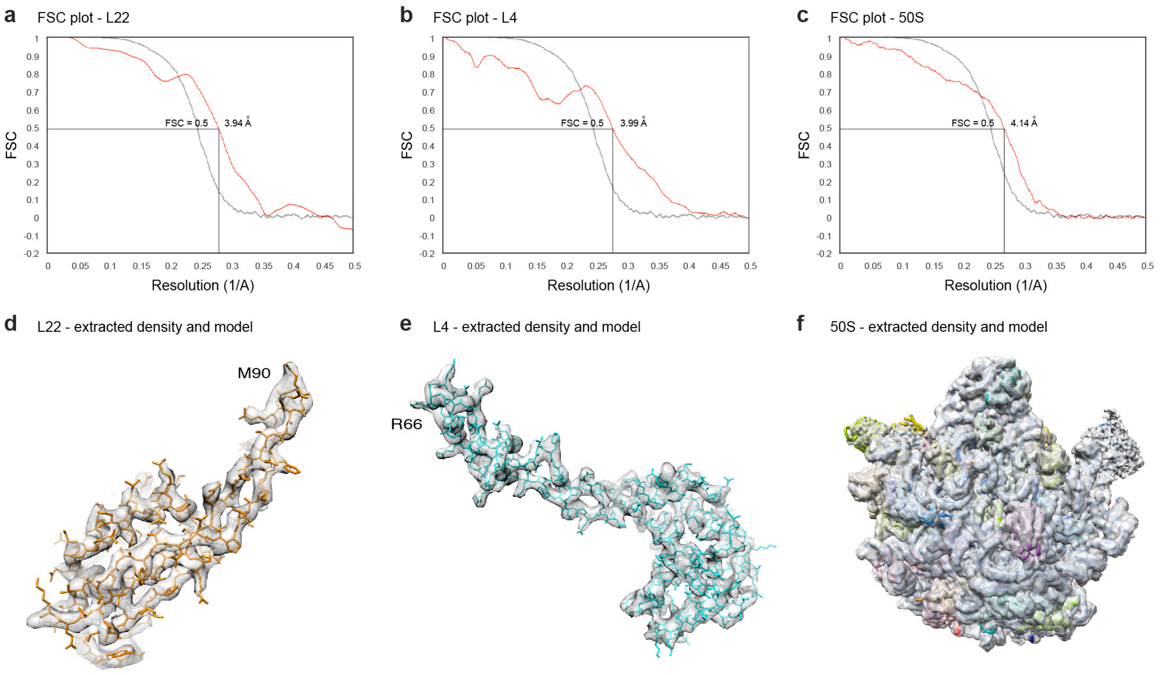

Supplementary Figure 5: Validation of the molecular model of the *B. subtilis* 50S subunit

a-c, Overlay of the FSC of the cryo-EM map of the MifM-SRC (black line from fig. S2) compared to the FSC calculation between the molecular model and cryo-EM map (red line) for **a**, L22, **b**, L4, and **c**, the complete *B. subtilis* 50S subunit. **d-f**, Fit of the molecular models into the cryo-EM map (grey mesh) of the MifM-SRC for **d**, L22 (orange), **e**, L4 (cyan) and the **f**, 50S subunit.

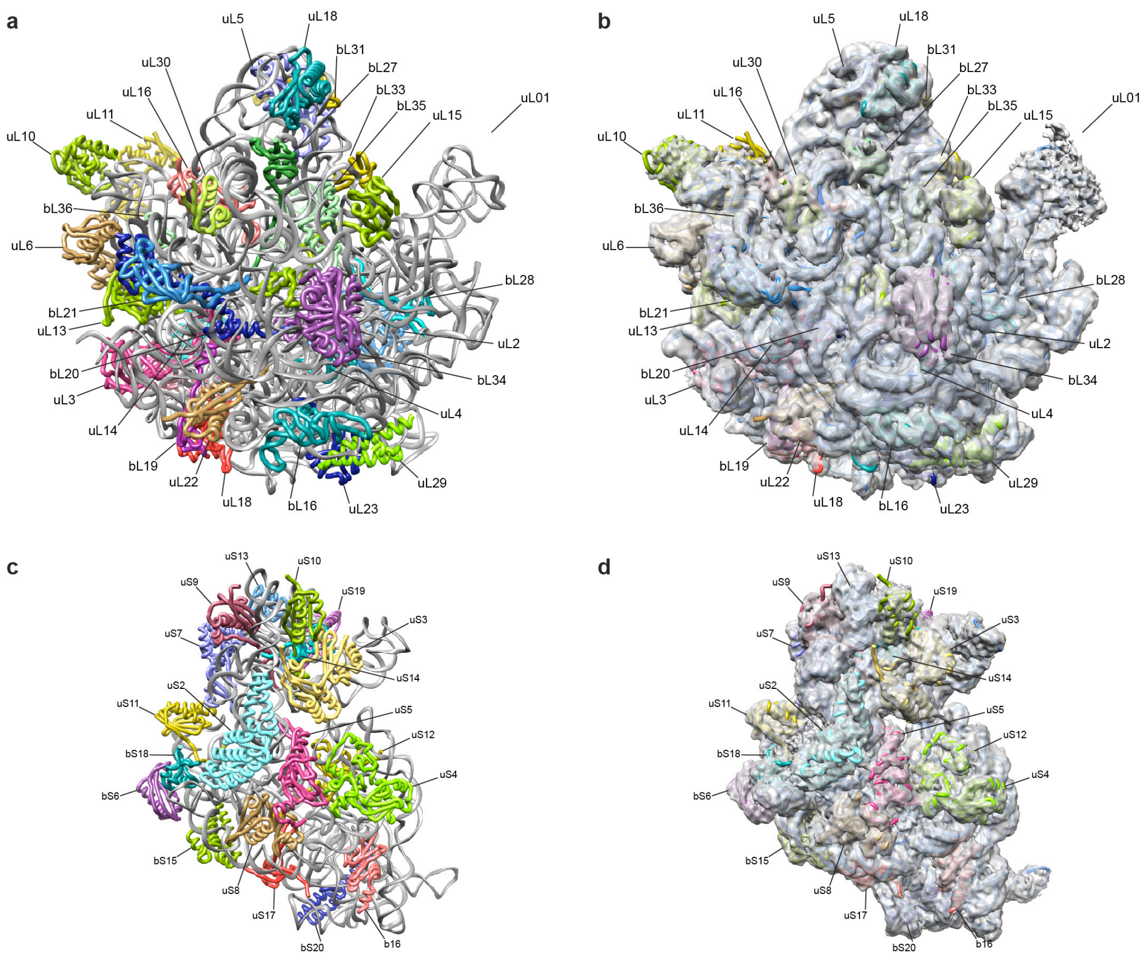

Supplementary Figure 6: Molecular model for the *Bacillus subtilis* 70S ribosome

a-d, Molecular model for the *B. subtilis* **a**,**b**, large 50S subunit and **c**,**d**, small 30S subunit. In **b**, and **d**, the molecular models are shown within the electron density (grey mesh) for the cryo-EM map of the MifM-SRC. Ribosomal proteins are coloured distinctly and labeled using the new nomenclature[6].

a

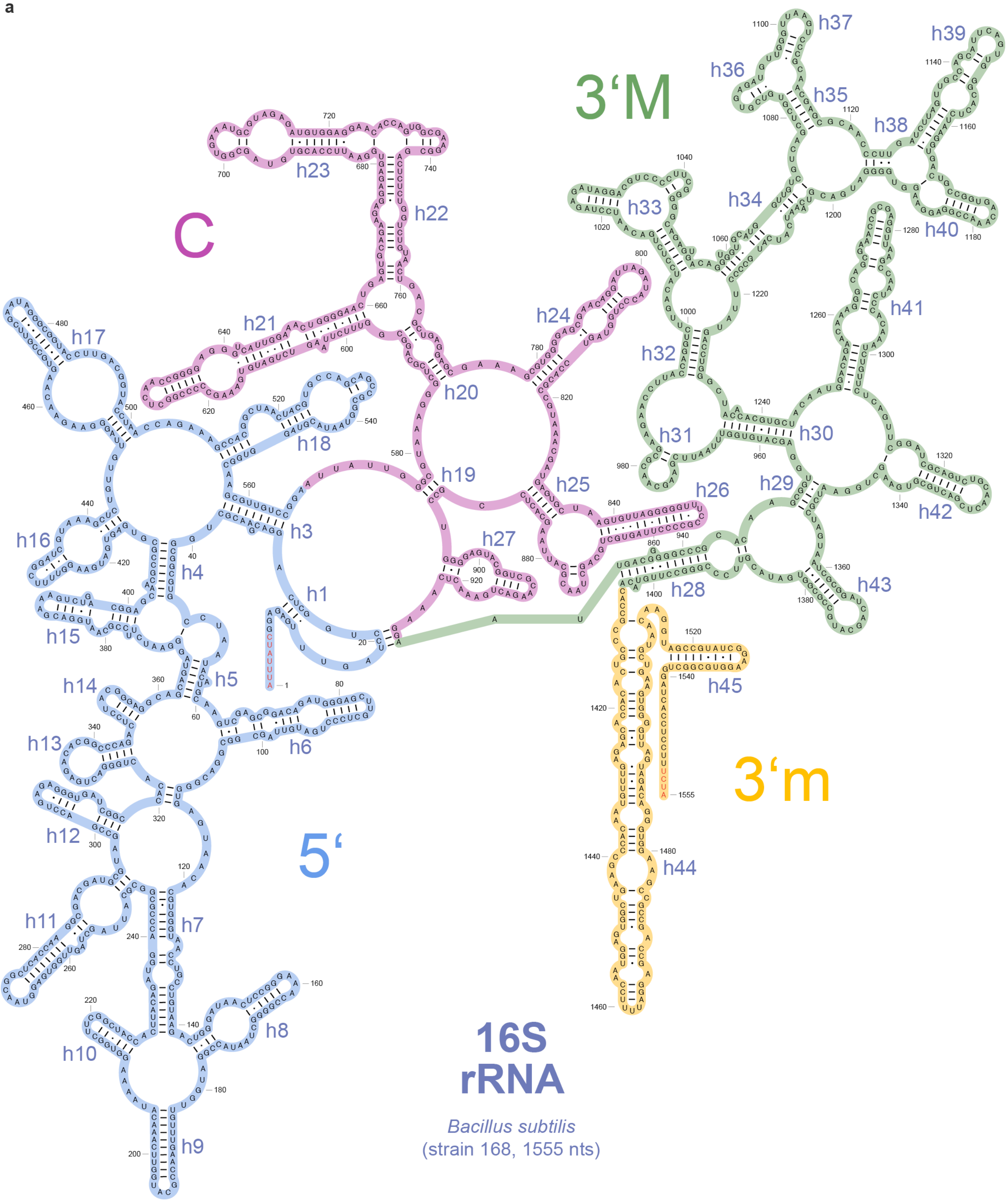

16S
rRNA
Bacillus subtilis
(strain 168, 1555 nts)
5‘
C
3‘M
3‘m
h1
h3
h4
h5
h6
h7
h8
h9
h10
h11
h12
h13
h14
h15
h16
h17
h18
h19
h20
h21
h22
h23
h24
h25
h26
h27
h28
h29
h30
h31
h32
h33
h34
h35
h36
h37
h38
h39
h40
h41
h42
h43
h44
h45

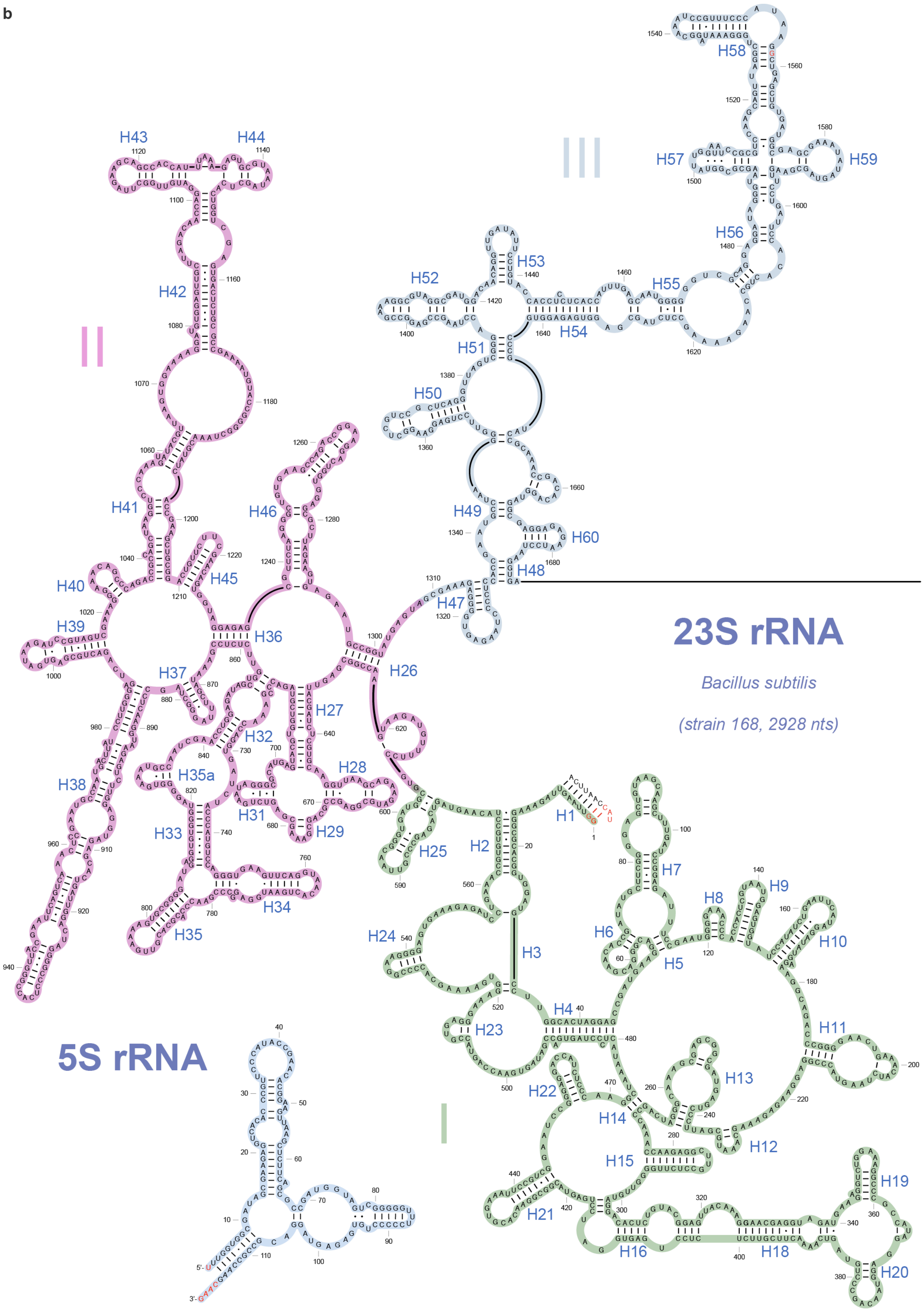
b
23S rRNA
Bacillus subtilis
(strain 168, 2928 nts)
5S rRNA
I
II
III
H1
H2
H3
H4
H5
H6
H7
H8
H9
H10
H11
H12
H13
H14
H15
H16
H18
H19
H20
H21
H22
H23
H24
H25
H26
H27
H28
H29
H31
H32
H33
H34
H35
H35a
H36
H37
H38
H39
H40
H41
H42
H43
H44
H45
H46
H47
H48
H49
H50
H51
H52
H53
H54
H55
H56
H57
H58
H59
H60

c

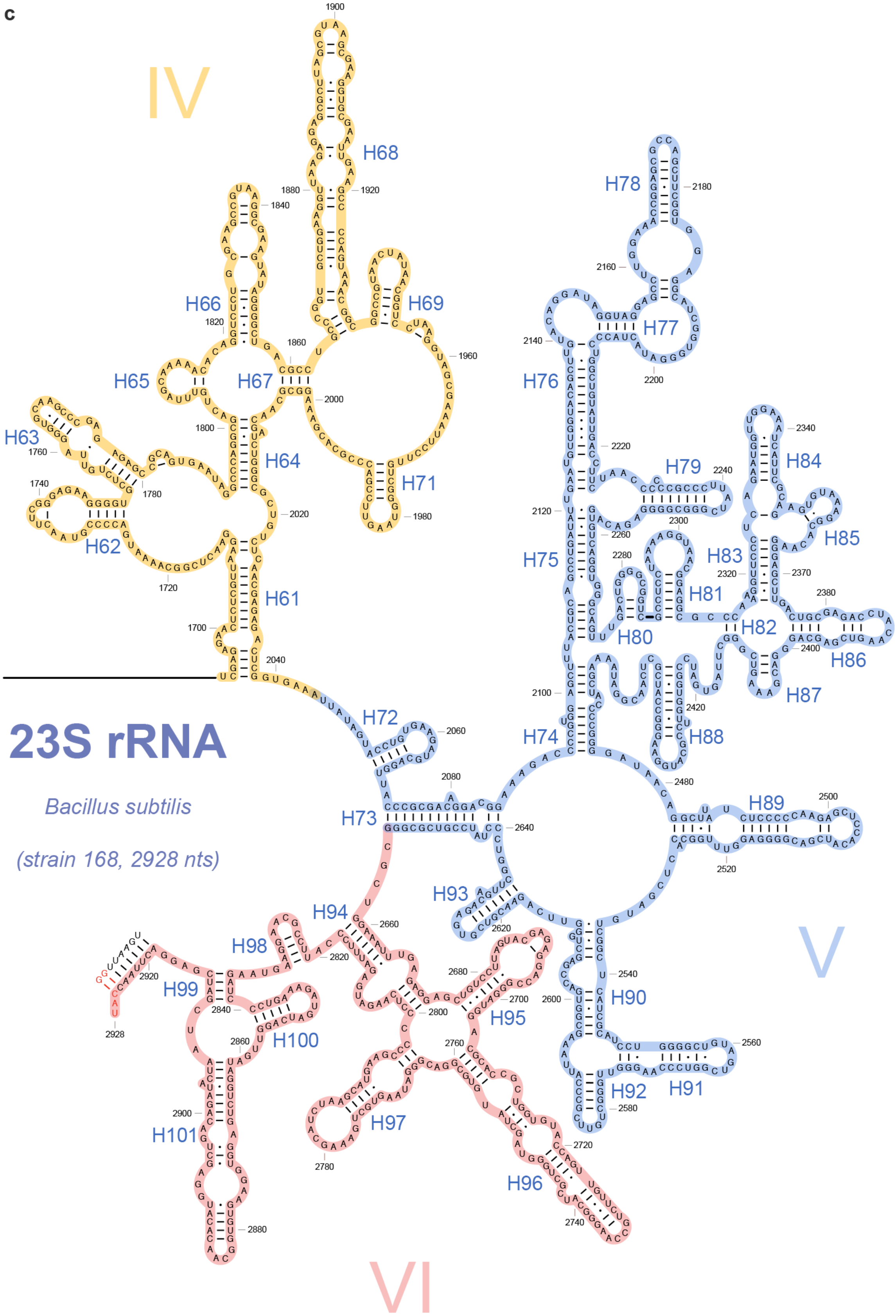
IV
V
VI
23S rRNA
Bacillus subtilis
(strain 168, 2928 nts)
H61
H62
H63
H64
H65
H66
H67
H68
H69
H71
H72
H73
H74
H75
H76
H77
H78
H79
H80
H81
H82
H83
H84
H85
H86
H87
H88
H89
H90
H91
H92
H93
H94
H95
H96
H97
H98
H99
H100
H101

Supplementary Figure 7: Secondary structures of the *B. subtilis* 5S, 16S and 23S rRNA.

a, Secondary structure of the *B. subtilis* 16S rRNA coloured according to the 5' (blue), central (C, magenta), 3' major (3'M, green) and 3' minor (3'm, yellow) domains. **b-c**, Secondary structure of the *B. subtilis* 5S and 23S rRNA coloured differently for domains I-VI. The secondary structures were modified from that available at the comparative RNA web (CRW) site[7] based on the molecular model of the *B. subtilis* 16S rRNA from the cryo-EM structure of the MifM-SRC.

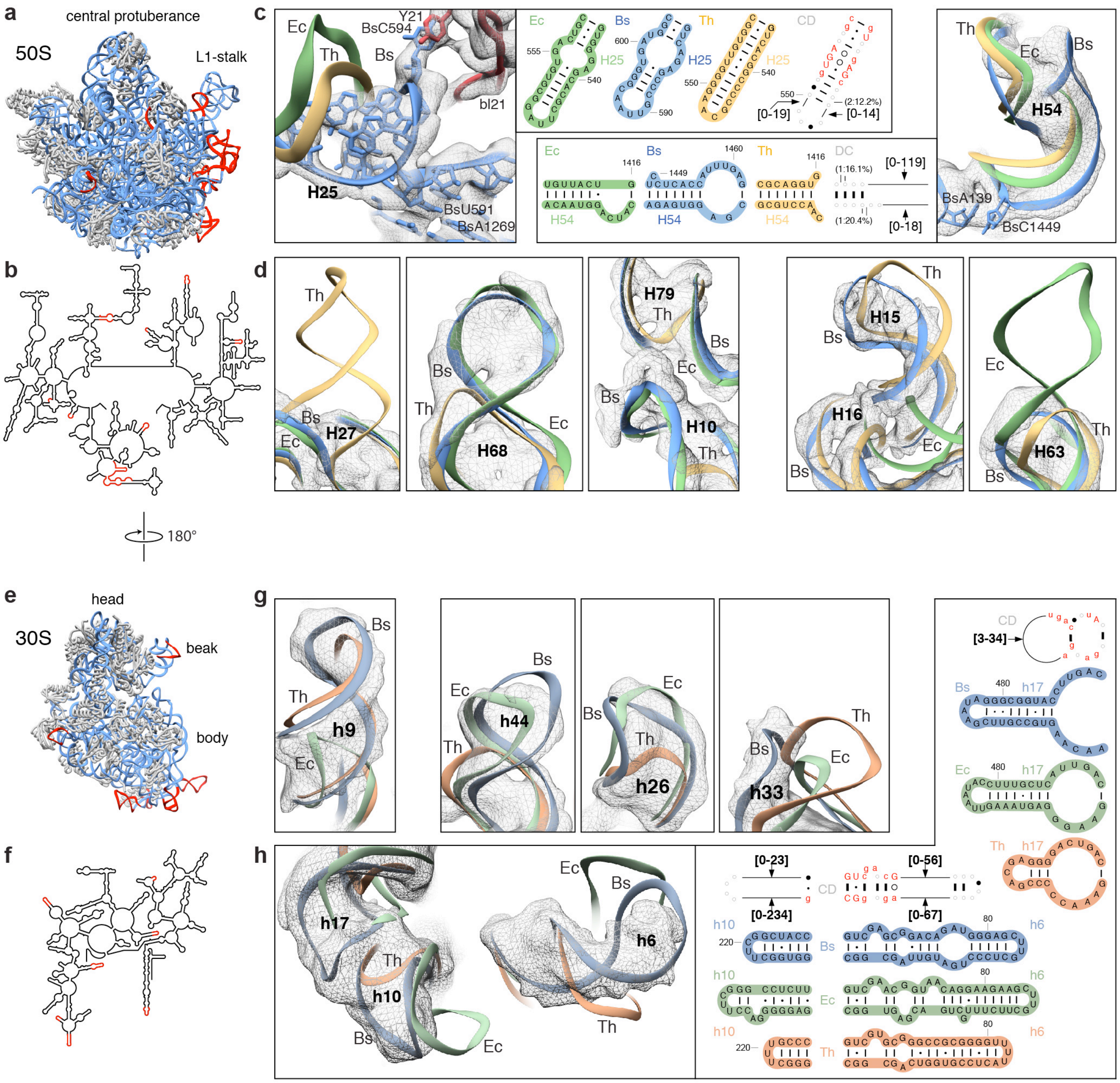

Supplementary Figure 8: Comparison of *B. subtilis*, *E. coli* and *T. thermophilus* 70S ribosome structures.

a-d, Regions of difference in 23S rRNA (red) mapped onto **a**, the *B. subtilis* 50S subunit (rRNA, blue and r-proteins, grey) and **b**, the secondary *B. subtilis* 23S rRNA. **c**, Panels show electron density (grey mesh) and rRNA models (blue) as well as secondary structures for regions of the *B. subtilis* 50S subunit that are distinct from the equivalent regions of the *E. coli* (green) and *T. thermophilus* (yellow) 50S subunit, namely H25 and H54. **d**, Panels show electron density (grey mesh) and rRNA models (blue) for regions of the *B. subtilis* 50S subunit that are either similar to *E. coli* (green, PDB4KIX/Y)[3, 4] and distinct from *T. thermophilus* (yellow, PDB3I8H/I)[5], for example H27, H68 and H10/H79, or similar to *T. thermophilus* and distinct

from *E. coli*, namely, H16 and H63. **e-h**, Regions of difference in 16S rRNA (red) mapped onto **e**, the *B. subtilis* 30S subunit (rRNA, blue and r-proteins, grey) and **f**, the secondary *B. subtilis* 16S rRNA. **g**, Panels show electron density (grey mesh) and rRNA models (blue) for regions of the *B. subtilis* 50S subunit that are either similar to *E. coli* and distinct from *T. thermophilus*, for example h9 or similar to *T. thermophilus* and distinct from *E. coli*, namely, h44, h26 and h33. **h**, Panels show electron density (grey mesh) and 16S rRNA models (blue) as well as secondary structures for regions of the *B. subtilis* 30S subunit that are distinct from the equivalent regions of the *E. coli* (green) and *T. thermophilus* (yellow) 30S subunit, namely h10/h17 and h6.

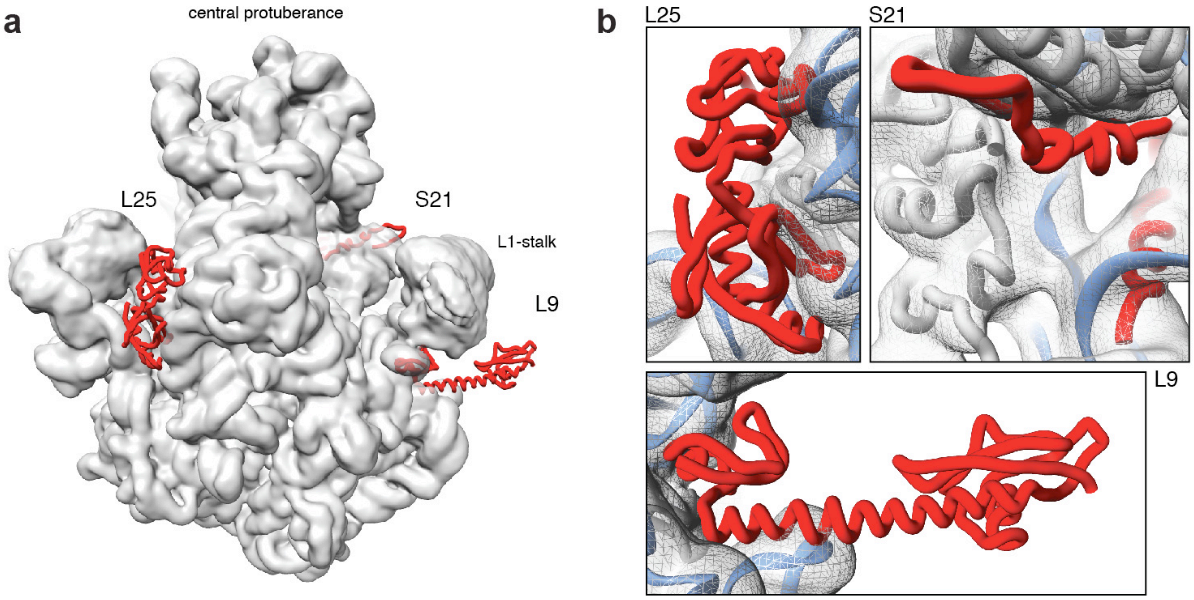

Supplementary Figure 9: Differences in ribosomal proteins between *B. subtilis*, *E. coli* and *T. thermophilus* ribosomes.

a, Overview and **b**, zoom of the cryo-EM map of the MifM-SRC (grey with map filtered to ~10 Å), showing the absence of density for ribosomal proteins S21, L9 and L25. The binding positions of S21 and L9 were taken from the *E. coli* (PDB4KIX/Y)[3, 4] and for L25 (red) from the *T. thermophilus* 70S ribosome (PDB3I8I)[5].

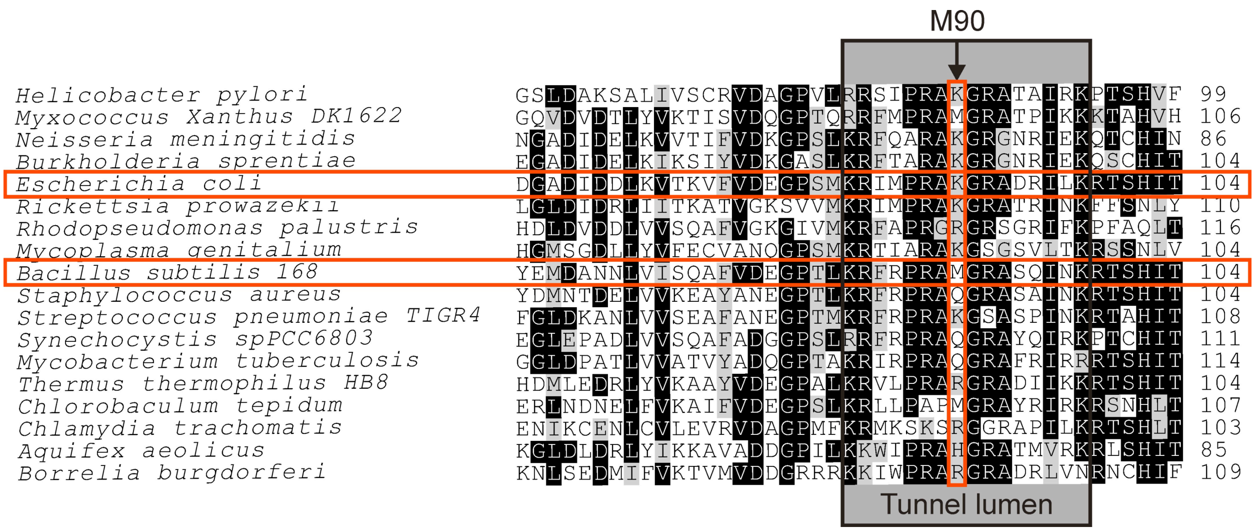

Supplementary Figure 10: Sequence alignment of the tunnel lumen region of bacterial ribosomal protein L22.

Boxshade representation of a Clustal W sequence alignment of selected bacterial ribosomal protein L22 sequences to highlight the sequence diversity at the position equivalent M90 in *B. subtilis* L22 (arrowed).

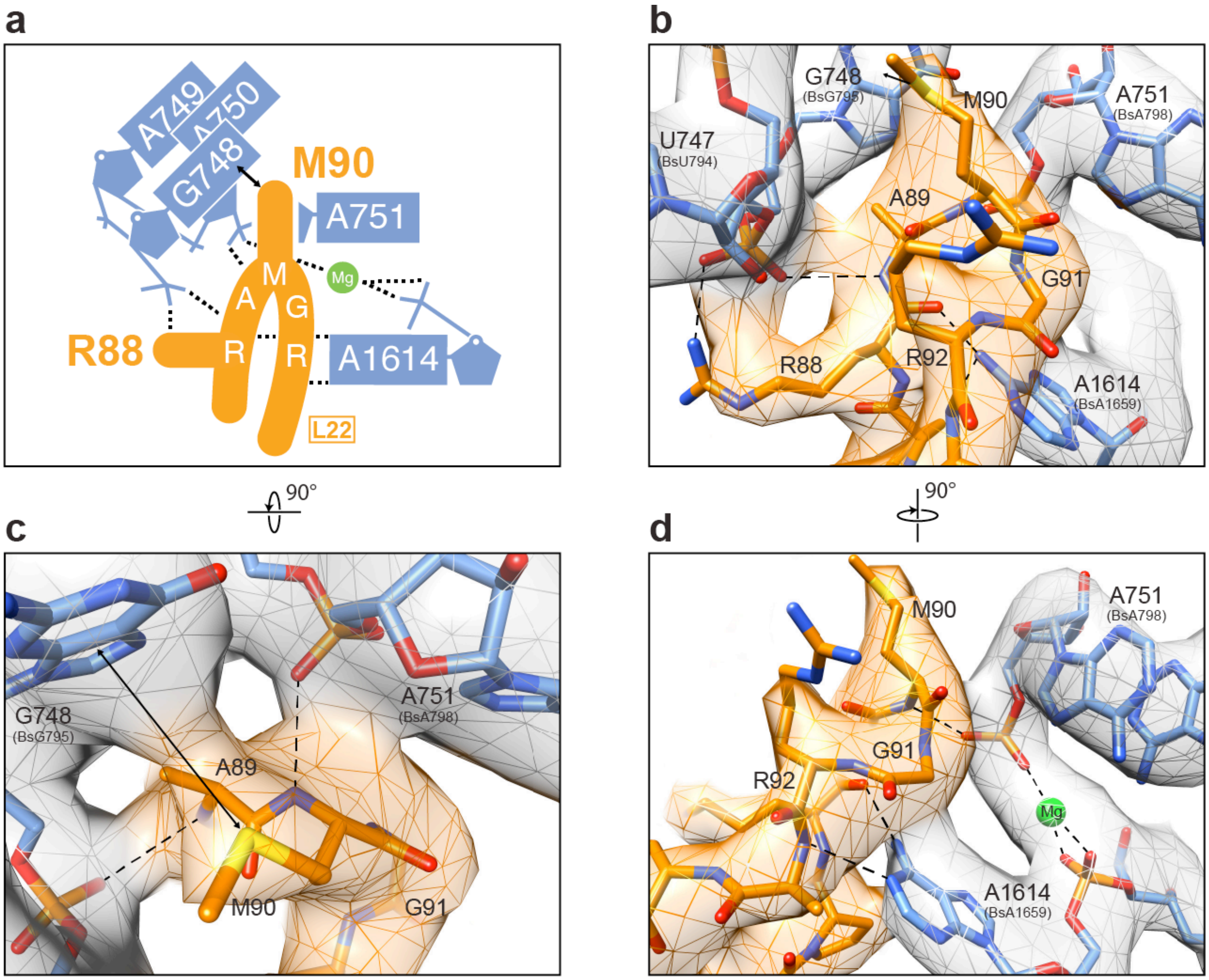

Supplementary Figure 11: Network of interactions between the tip of the β-hairpin of L22 and H35 of the 23S rRNA.

a, Schematic illustrating the network of interactions between the tip of the β-hairpin of L22 and the residues in H35 of the 23S rRNA. **b**, The backbone and the side chain of R88 of L22 are within hydrogen bonding distance to the phosphate-oxygens of G748. **c**, M90 of L22 can form sulphur-Pi interaction with the nucleobase of G748, whereas the backbone nitrogen of M90 is within hydrogen bonding distance to the phosphate-oxygen of A751. **d**, The backbone phosphate-oxygens of A751 and A1614 coordinate a Mg^{2+} ion, while the nucleobase of A1614 comes within hydrogen bonding distance of the backbone of A89 and R92 of L22.

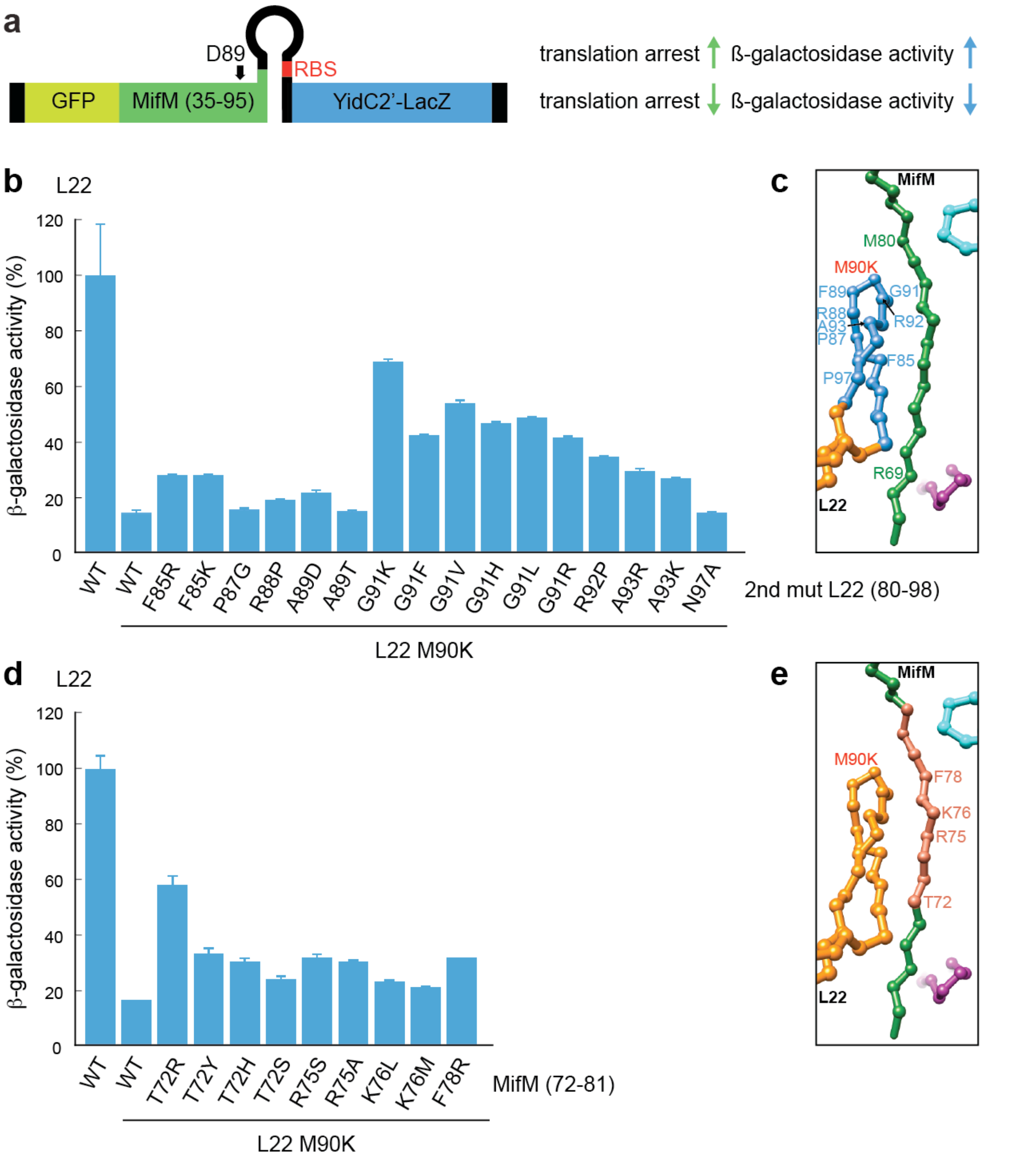

Supplementary Figure 12: Identification of second site mutations in MifM or L22 that restore stalling in the context of the L22-M90K mutation. a, Schematic for the GFP-MifM-YidC2'-LacZ induction reporter used to monitor translational arrest via β-galactosidase activity in *B. subtilis in vivo*. In contrast to the GFP-MifM-LacZ reporter (see Figure 3g), translational arrest using the GFP-MifM-YidC2'-LacZ reporter leads to induction of β-galactosidase activity. **b**, β-galactosidase activity from the GFP-MifM-YidC2'-LacZ reporter using *B. subtilis* strains

bearing the *B. subtilis* L22-M90K mutation selected for second site suppressor mutations within L22 (residues 80-98) that restore stalling and induction of β-galactosidase activity. **c**, Overview of relative positions of MifM to tunnel lumen residues of L22 that rescue the L22-M90K mutation. **d**, β-galactosidase activity from the GFP-MifM-YidC2'-LacZ reporter when using *B. subtilis* strains bearing the *B. subtilis* L22-M90K mutation selected for second site suppressors within MifM (residues 72-81) that restore stalling and induction of β-galactosidase activity. **e**, Overview of relative positions of tunnel lumen residues of L22 to MifM residues that rescue the L22-M90K mutation. In **b** and **d**, the error bars indicate the standard deviation of three independent biological replicates.

Supplementary Table 1: Strains	
Strains	genotype
BKG80	*amyE::rbsm1-gfp-mifM*$^{35\text{-}95}$*-lacZΩcat, rpsSΩkanΩrplV(M90C)*
NAB263	*amyE::rbsm1-gfp-mifM*$^{35\text{-}95}$*-lacZΩcat, rpsSΩkanΩrplV(M90V)*
NAB264	*amyE::rbsm1-gfp-mifM*$^{35\text{-}95}$*-lacZΩcat, rpsSΩkanΩrplV(M90D)*
NAB265	*amyE::rbsm1-gfp-mifM*$^{35\text{-}95}$*-lacZΩcat, rpsSΩkanΩrplV(M90F)*
NAB269	*amyE::rbsm1-gfp-mifM*$^{35\text{-}95}$*-lacZΩcat, rpsSΩkanΩrplV(M90S)*
NAB270	*amyE:: rbsm1-gfp-mifM*$^{35\text{-}95}$*-lacZΩcat, rpsSΩkanΩrplV(M90N)*
NAB272	*amyE::rbsm1-gfp-mifM*$^{35\text{-}95}$*-lacZΩcat, rpsSΩkanΩrplV(M90L)*
NAB275	*amyE::rbsm1-gfp-mifM*$^{35\text{-}95}$*-lacZΩcat, rpsSΩkanΩrplV(M90I)*
NAB276	*amyE::rbsm1-gfp-mifM*$^{35\text{-}95}$*-lacZΩcat, rpsSΩkanΩrplV(M90R)*
NAB277	*amyE::rbsm1-gfp-mifM*$^{35\text{-}95}$*-lacZΩcat, rpsSΩkanΩrplV(M90E)*
NAB278	*amyE::rbsm1-gfp-mifM*$^{35\text{-}95}$*-lacZΩcat, rpsSΩkanΩrplV(M90A)*
NAB279	*amyE::rbsm1-gfp-mifM*$^{35\text{-}95}$*-lacZΩcat, rpsSΩkanΩrplV(M90T)*
NAB280	*amyE::rbsm1-gfp-mifM*$^{35\text{-}95}$*-lacZΩcat, rpsSΩkanΩrplV(M90Q)*
NAB281	*amyE::rbsm1-gfp-mifM*$^{35\text{-}95}$*-lacZΩcat, rpsSΩkanΩrplV(M90Y)*
NAB285	*amyE::rbsm1-gfp-mifM*$^{35\text{-}95}$*-lacZΩcat, rpsSΩkanΩrplV(M90G)*
NAB286	*amyE::rbsm1-gfp-mifM*$^{35\text{-}95}$*-lacZΩcat, rpsSΩkanΩrplV(M90P)*
NAB287	*amyE::rbsm1-gfp-mifM*$^{35\text{-}95}$*-lacZΩcat, rpsSΩkanΩrplV(M90W)*
NAB291	*amyE::rbsm1-gfp-mifM*$^{35\text{-}95}$*-lacZΩcat, rpsSΩkanΩrplV(M90H)*
SCB824	*amyE::gfp-mifM*$^{35\text{-}95}$*-yidC2'-lacZΩcat*
SCB2592	*amyE::rbsm1-gfp-mifM*$^{35\text{-}95}$*-lacZΩcat*
SCB2619	*rplWΩkanΩrplB*
SCB2634	*rplW(d65-69)Ωkan*
SCB2656	*rpsSΩkan*
SCB2939	*rplD(d63-67)Ωkan*
SCB2942	*rplD(d66-70)Ωkan*
SCB2912	*rpsSΩkanΩrplV(d82-86)*
SCB2917	*rpsSΩkanΩrplV(d86-90)*
SCB2920	*rpsSΩkanΩrplV(d91-95)*
SCB3348	*rpsSΩkanΩrplV(M90K)*
SCB2639	*amyE::rbsm1-gfp-mifM*$^{35\text{-}95}$*-lacZΩcat, rplWΩkan*
SCB2639	*amyE::rbsm1-gfp-mifM*$^{35\text{-}95}$*-lacZΩcat, rplWΩkan*
SCB2655	*amyE::rbsm1-gfp-mifM*$^{35\text{-}95}$*-lacZΩcat, rplW(d65-69)Ωkan*
SCB2924	*amyE::gfp-mifM*$^{35\text{-}95}$*-yidC2'-lacZΩcat, rpsSΩkanΩrplV*

SCB2956 *amyE::rbsm1-gfp-mifM*$^{35-95}$*-lacZΩcat, rplD(d63-67)Ωkan*

SCB2957 *amyE::rbsm1-gfp-mifM*$^{35-95}$*-lacZΩcat, rplD(d66-70)Ωkan*

SCB2958 *amyE::rbsm1-gfp-mifM*$^{35-95}$*-lacZΩcat, rpsSΩkanΩrplV*

SCB2959 *amyE::rbsm1-gfp-mifM*$^{35-95}$*-lacZΩcat, rpsSΩkanΩrplV(d82-86)*

SCB2960 *amyE::rbsm1-gfp-mifM*$^{35-95}$*-lacZΩcat, rpsSΩkanΩrplV(d86-90)*

SCB2961 *amyE::rbsm1-gfp-mifM*$^{35-95}$*-lacZΩcat, rpsSΩkanΩrplV(d91-95)*

SCB3247 *amyE::rbsm1-gfp-mifM*$^{35-95}$*-lacZΩcat, rpsSΩkanΩrplV(M90K)*

SCB3251 *amyE::rbsm1-gfp-mifM*$^{35-95}$*-lacZΩcat, rpsSΩkanΩrplV(Ec-tip)*

SCB3272 *amyE::rbsm1-gfp-mifM*$^{35-95}$*-lacZΩcat, rpsSΩkanΩrplV(Ec-tip)-I85F/M86R*

SCB3274 *amyE::rbsm1-gfp-mifM*$^{35-95}$*-lacZΩcat, rpsSΩkanΩrplV(Ec-tip)-K90M*

SCB3353 *amyE::gfp-mifM*$^{35-95}$*-yidC2'-lacZΩcat, rpsSΩkanΩrplV(M90K)*

SCB3353 *amyE::gfp-mifM*$^{35-95}$*-yidC2'-lacZΩcat, rpsSΩkanΩrplV(M90K)*

SCB3378 *amyE::gfp-mifM*$^{35-95}$*-yidC2'-lacZΩcat, rplV94*

SCB3523 *amyE::gfp-mifM(T72H)*$^{35-95}$*-yidC2'-lacZΩcat, rpsSΩkanΩrplV(M90K)*

SCB3524 *amyE::gfp-mifM(T72Y)*$^{35-95}$*-yidC2'-lacZΩcat, rpsSΩkanΩrplV(M90K)*

SCB3525 *amyE::gfp-mifM(T72S)*$^{35-95}$*-yidC2'-lacZΩcat, rpsSΩkanΩrplV(M90K)*

SCB3527 *amyE::gfp-mifM(R75S)*$^{35-95}$*-yidC2'-lacZΩcat, rpsSΩkanΩrplV(M90K)*

SCB3529 *amyE::gfp-mifM(K76M)*$^{35-95}$*-yidC2'-lacZΩcat, rpsSΩkanΩrplV(M90K)*

SCB3530 *amyE::gfp-mifM(K76L)*$^{35-95}$*-yidC2'-lacZΩcat, rpsSΩkanΩrplV(M90K)*

SCB3532 *amyE::gfp-mifM(F78R)*$^{35-95}$*-yidC2'-lacZΩcat, rpsSΩkanΩrplV(M90K)*

SCB3533 *amyE::gfp-mifM(R75A)*$^{35-95}$*-yidC2'-lacZΩcat, rpsSΩkanΩrplV(M90K)*

SCB3572 *amyE::gfp-mifM*$^{35-95}$*-yidC2'-lacZΩcat, rpsSΩkanΩrplV(M90K/F85R)*

SCB3573 *amyE::gfp-mifM*$^{35-95}$*-yidC2'-lacZΩcat, rpsSΩkanΩrplV(M90K/F85K)*

SCB3574 *amyE::gfp-mifM*$^{35-95}$*-yidC2'-lacZΩcat, rpsSΩkanΩrplV(M90K/P87G)*

SCB3575 *amyE::gfp-mifM*$^{35-95}$*-yidC2'-lacZΩcat, rpsSΩkanΩrplV(M90K/R88P)*

SCB3576 *amyE::gfp-mifM*$^{35-95}$*-yidC2'-lacZΩcat, rpsSΩkanΩrplV(M90K/A89D)*

SCB3577 *amyE::gfp-mifM*$^{35-95}$*-yidC2'-lacZΩcat, rpsSΩkanΩrplV(M90K/A89T)*

SCB3578 *amyE::gfp-mifM*$^{35-95}$*-yidC2'-lacZΩcat, rpsSΩkanΩrplV(M90K/G91K)*

SCB3579 *amyE::gfp-mifM*$^{35-95}$*-yidC2'-lacZΩcat, rpsSΩkanΩrplV(M90K/G91L)*

SCB3580 *amyE::gfp-mifM*$^{35-95}$*-yidC2'-lacZΩcat, rpsSΩkanΩrplV(M90K/G91F)*

SCB3581 *amyE::gfp-mifM*$^{35-95}$*-yidC2'-lacZΩcat, rpsSΩkanΩrplV(M90K/G91H)*

SCB3582 *amyE::gfp-mifM*$^{35-95}$*-yidC2'-lacZΩcat, rpsSΩkanΩrplV(M90K/G91Y)*

SCB3583 *amyE::gfp-mifM*$^{35-95}$*-yidC2'-lacZΩcat, rpsSΩkanΩrplV(M90K/R92P)*

SCB3584 *amyE::gfp-mifM*$^{35-95}$*-yidC2'-lacZΩcat, rpsSΩkanΩrplV(M90K/A93R)*

SCB3585 *amyE::gfp-mifM*$^{35-95}$*-yidC2'-lacZΩcat, rpsSΩkanΩrplV(M90K/A93K)*

SCB3586 *amyE::gfp-mifM*$^{35-95}$*-yidC2'-lacZΩcat, rpsSΩkanΩrplV(M90K/G91R)*

SCB3605	*amyE::gfp-mifM*$^{35\text{-}95}$*-yidC2'-lacZΩcat, rpsSΩkanΩrplV(M90K/N97A)*
SCB3607	*amyE::gfp-mifM(T72R)*$^{35\text{-}95}$*-yidC2'-lacZΩcat, rpsSΩkanΩrplV(M90K)*
SCB3610	*amyE::gfp-mifM(T72R)*$^{35\text{-}95}$*-yidC2'-lacZΩcat, rpsSΩkanΩrplV*

**Supplementary Table 2: Plasmids**				
plasmid	genotype	Primer	Template	ref
pCH735	*amyE::mifM-lacZΩcat*			[8]
pCH835	*amyE::GFP-mifM*$^{35\text{-}95}$*-yidC2'-lacZΩcat*			[8]
pCH913	*amyE::rbsm1-GFP-mifM*$^{35\text{-}95}$*-yidC2'-lacZΩcat*			[8]
pCH1142	*spcRΩloxP-kanR-loxP*			[9]
pCH1517	*amyE::rbsm1-GFP-mifM*$^{35\text{-}95}$*-lacZΩcat*			This study
pCH1570	*rplWΩkanΩrplB*			This study
pCH1584	*rplW(d65-69)ΩkanΩrplB*	SP1	pCH1570	This study
pCH1587	*rpsSΩkanΩrplV*			This study
pCH1590	*rpsSΩkanΩrplV(d82-86)*	SP2	pCH1587	This study
pCH1591	*rpsSΩkanΩrplV(d86-90)*	SP3	pCH1587	This study
pCH1592	*rpsSΩkanΩrplV(d91-95)*	SP4	pCH1587	This study
pCH1745	*spcRΩrplCD(d63-67)WΩkanΩrplB*	SP5	pCH1744	This study
pCH1746	*spcRΩrplCD(d66-70)WΩkanΩrplB*	SP6	pCH1744	This study
pCH1897	*rpsSΩkanΩrplV(M90K)*	SP7	pCH1587	This study
pCH1901	*rpsSΩkanΩrplV(Ec-tip)*			This study
pCH1904	*rpsSΩkanΩrplV(Ec-tip)-I85F-M86R*	SP8	pCH1901	This study
pCH1905	*rpsSΩkanΩrplV(Ec-tip)-K90M*	SP9	pCH1901	This study
pCH1958	*amyE::GFP-mifM(T72H)*$^{35\text{-}95}$*-yidC2'-lacZΩcat*	SP10	pCH835	This study
pCH1959	*amyE::GFP-mifM(T72Y)*$^{35\text{-}95}$*-yidC2'-lacZΩcat*	SP11	pCH835	This study
pCH1960	*amyE::GFP-mifM(T72S)*$^{35\text{-}95}$*-yidC2'-lacZΩcat*	SP12	pCH835	This study
pCH1962	*amyE::GFP-mifM(R75S)*$^{35\text{-}95}$*-yidC2'-lacZΩcat*	SP13	pCH835	This study
pCH1964	*amyE::GFP-mifM(K76M)*$^{35\text{-}95}$*-yidC2'-lacZΩcat*	SP14	pCH835	This study
pCH1965	*amyE::GFP-mifM(K76L)*$^{35\text{-}95}$*-yidC2'-lacZΩcat*	SP15	pCH835	This study
pCH1967	*amyE::GFP-mifM(F78R)*$^{35\text{-}95}$*-yidC2'-lacZΩcat*	SP16	pCH835	This study
pCH1968	*amyE::GFP-mifM(R75A)*$^{35\text{-}95}$*-yidC2'-lacZΩcat*	SP17	pCH835	This study
pCH1978	*amyE::GFP-mifM(T72R)*$^{35\text{-}95}$*-yidC2'-lacZΩcat*	SP18	pCH835	This study
pEB71	*loxP-kanR-loxP*			[10]
pKG11	*rpsSΩkanΩrplV(M90C)*	SP19	pCH1587	This study
pNAR479	*rpsSΩkanΩrplV(M90V)*	SP20	pCH1587	This study
pNAR483	*rpsSΩkanΩrplV(M90D)*	SP20	pCH1587	This study
pNAR484	*rpsSΩkanΩrplV(M90F)*	SP20	pCH1587	This study
pNAR485	*rpsSΩkanΩrplV(M90S)*	SP20	pCH1587	This study

pNAR487	*rpsSΩkanΩrplV(M90N)*	SP20	pCH1587	This study
pNAR489	*rpsSΩkanΩrplV(M90L)*	SP20	pCH1587	This study
pNAR490	*rpsSΩkanΩrplV(M90I)*	SP20	pCH1587	This study
pNAR491	*rpsSΩkanΩrplV(M90R)*	SP20	pCH1587	This study
pNAR494	*rpsSΩkanΩrplV(M90E)*	SP20	pCH1587	This study
pNAR495	*rpsSΩkanΩrplV(M90A)*	SP20	pCH1587	This study
pNAR496	*rpsSΩkanΩrplV(M90T)*	SP20	pCH1587	This study
pNAR498	*rpsSΩkanΩrplV(M90Q)*	SP20	pCH1587	This study
pNAR499	*rpsSΩkanΩrplV(M90Y)*	SP21	pCH1587	This study
pNAR500	*rpsSΩkanΩrplV(M90G)*	SP22	pCH1587	This study
pNAR501	*rpsSΩkanΩrplV(M90P)*	SP23	pCH1587	This study
pNAR502	*rpsSΩkanΩrplV(M90W)*	SP24	pCH1587	This study
pNAR503	*rpsSΩkanΩrplV(M90H)*	SP25	pCH1587	This study

Supplementary Table 3: Primers

primer	sequence
MifMfor	5'- GAAATTAATACGACTCACTATAGGG -3'
MifMrev	5'- TTATTATTATTAGTCTTCCTCATCGTTCACAGG -3'
SP1	5'-ACTACAAAGGCAAATCAAAAACTGGTATGACTAGCCGTCG-3'
SP2	5'-TCGTTGACGAAGGCCCTACGCCACGTGCTATGGGACGTGC-3'
SP3	5'-GCCCTACGTTAAAAAGATTCGGACGTGCGAGCCAAATCAA-3'
SP4	5'-GATTCCGCCCACGTGCTATGATCAACAAACGTACGAGCCA-3'
SP5	5'-AAGTACGCGGCGGAGGTCGTAAAGGTACTGGACGTGCCCG-3'
SP6	5'-GCGGAGGTCGTAAACCATGGGGACGTGCCCGTCAAGGTTC-3'
SP7	5'-AGATTCCGCCCACGTGCTAAAGGACGTGCGAGCCAAATC-3'
SP8	5'-GGCCCTAGCATGAAGCGCTTCCGCCCGCGTGCAAAAGGTCGT-3'
SP9	5'-CGCATTATGCCGCGTGCAATGGGTCGTGCAGATCGCATC-3'
SP10	5'-ATATATCATCGCATTACACATTGGATACGTAAAGTCTTC-3'
SP11	5'-ATATATCATCGCATTACATATTGGATACGTAAAGTCTTC-3'
SP12	5'-ATATATCATCGCATTACAAGCTGGATACGTAAAGTCTTC-3'
SP13	5'-CGCATTACAACTTGGATAAGCAAAGTCTTCCGCATGAAT-3'
SP14	5'-ATTACAACTTGGATACGTATGGTCTTCCGCATGAATTCG-3'
SP15	5'-ATTACAACTTGGATACGTCTGGTCTTCCGCATGAATTCG-3'
SP16	5'-ACTTGGATACGTAAAGTCCGCCGCATGAATTCGCCTGTG-3'
SP17	5'-CGCATTACAACTTGGATAGCGAAAGTCTTCCGCATGAA-3'
SP18	5'-ATATATCATCGCATTACACGCTGGATACGTAAAGTCTTC-3'
SP19	5'-AGATTCCGCCCACGTGCTTGCGGACGTGCGAGCCAAATC-3'
SP20	5'-AGATTCCGCCCACGTGCTNNNGGACGTGCGAGCCAAATC-3'
SP21	5'-AGATTCCGCCCACGTGCTTATGGACGTGCGAGCCAAATC-3'
SP22	5'-AGATTCCGCCCACGTGCTGGCGGACGTGCGAGCCAAATC-3'
SP23	5'-AGATTCCGCCCACGTGCTCCGGGACGTGCGAGCCAAATC-3'
SP24	5'-AGATTCCGCCCACGTGCTTGGGGACGTGCGAGCCAAATC-3'
SP25	5'-AGATTCCGCCCACGTGCTCATGGACGTGCGAGCCAAATC-3'
SP26	5'-CAAGGAATGGTGCATGCAAGG-3'
SP27	5'-TCCATAGATCTTAAAAGAAGAGAACCGGCGTC-3'
SP28	5'-AATAAGAGCTCCAGGTTCTTGGTAAAGAAGGT-3'
SP29	5'-TCCTCTTTTCTACAGTATTTAGGATCCTTAGCGTCTTGTTTTTTTGTC-3'
SP30	5'-TTTTACTGGATGAATTGTTTTAGCTCGAGTAATTAAACAAGACGCTAAGAGAGGA-3'
SP31	5'-TTATTGCATGCTCTTCAGACACACCAGAAATT-3'

SP32	5'-TAAGGATCCTAAATACTGTAGAAAAGAGGA-3'
SP33	5'-AATTACTCGAGCTAAAACAATTCATCCAGTAAAA-3'
SP34	5'-GGGAAGAACAGTATGTCGAGC-3'
SP35	5'-CTGCACGACCTTTTGCACGCGGCATAATGCGCTTCATGCTAGGGCCTTCGTCAACGAATGC-3'
SP36	5'-GCATTATGCCGCGTGCAAAAGGTCGTGCAGATCGCATCCTGAAACGTACGAGCCACATTACA-3'
SP37	5'-TGTGGAATTGTGAGCGG-3'
SP38	5'-GCATTCGTTGACGAAGGCNNNACGTTAAAAAGATTCCGC-3'
SP39	5'-TTCGTTGACGAAGGCCCTNNNTTAAAAAGATTCCGCCCA-3'
SP40	5'-GTTGACGAAGGCCCTACGNNNAAAAGATTCCGCCCACGT-3'
SP41	5'-GACGAAGGCCCTACGTTANNNAGATTCCGCCCACGTGCT-3'
SP42	5'-GAAGGCCCTACGTTAAAANNNTTCCGCCCACGTGCTAAA-3'
SP43	5'-GGCCCTACGTTAAAAAGANNNCGCCCACGTGCTAAAGGA-3'
SP44	5'-CCTACGTTAAAAAGATTCNNNCCACGTGCTAAAGGACGT-3'
SP45	5'-ACGTTAAAAAGATTCCGCNNNCGTGCTAAAGGACGTGCG-3'
SP46	5'-TTAAAAAGATTCCGCCCANNNGCTAAAGGACGTGCGAGC-3'
SP47	5'-AAAAGATTCCGCCCACGTNNNAAAGGACGTGCGAGCCAA-3'
SP48	5'-TTCCGCCCACGTGCTAAANNNCGTGCGAGCCAAATCAAC-3'
SP49	5'-CGCCCACGTGCTAAAGGANNNGCGAGCCAAATCAACAAA-3'
SP50	5'-CCACGTGCTAAAGGACGTNNNAGCCAAATCAACAAACGT-3'
SP51	5'-CGTGCTAAAGGACGTGCGNNNCAAATCAACAAACGTACG-3'
SP52	5'-GCTAAAGGACGTGCGAGCNNNATCAACAAACGTACGAGC-3'
SP53	5'-AAAGGACGTGCGAGCCAANNNAACAAACGTACGAGCCAC-3'
SP54	5'-GGACGTGCGAGCCAAATCNNNAAACGTACGAGCCACATT-3'
SP55	5'-CGTGCGAGCCAAATCAACNNNCGTACGAGCCACATTACA-3'
SP56	5'-ATATATCATCGCATTACANNNTGGATACGTAAAGTCTTC-3'
SP57	5'-TATCATCGCATTACAACTNNNATACGTAAAGTCTTCCGC-3'
SP58	5'-CATCGCATTACAACTTGGNNNCGTAAAGTCTTCCGCATG-3'
SP59	5'-CGCATTACAACTTGGATANNNAAAGTCTTCCGCATGAAT-3'
SP60	5'-ATTACAACTTGGATACGTNNNGTCTTCCGCATGAATTCG-3'
SP61	5'-ACAACTTGGATACGTAAANNNTTCCGCATGAATTCGCCT-3'
SP62	5'-ACTTGGATACGTAAAGTCNNNCGCATGAATTCGCCTGTG-3'
SP63	5'-TGGATACGTAAAGTCTTCNNNATGAATTCGCCTGTGAAC-3'
SP64	5'-ATACGTAAAGTCTTCCGCNNNAATTCGCCTGTGAACGAT-3'
SP65	5'-CGTAAAGTCTTCCGCATGNNNTCGCCTGTGAACGATGAG-3'
SP66	5'-TTTAGTCTAGATAATTATCTGTAAAAAGAAGG-3'
SP67	5'-ATATTGCATGCACCACGAACTGTAGGACGGAT-3'

SP68	5'-AATAAGGATCCCCAAAACGGTTCTACTGCTGG-3'
SP69	5'-TTAAGCTTCAAAAATTTCGAT-3'
SP70	5'-GAAATCGAAATTTTTGAAGCTTAAATACTGTAGAAAAGAGGAAGG-3'
SP71	5'-CTAAAACAATTCATCCAGTAAAA-3'
SP72	5'-AATTATCTAGACTAAAACAATTCATCCAGTAAAA-3'

‘N’ represents mixture of A, G, T and C.

Supplementary References:

1. Scheres SH, Chen S. Prevention of overfitting in cryo-EM structure determination. *Nat Methods* **9**, 853-854 (2012).

2. Kucukelbir A, Sigworth FJ, Tagare HD. Quantifying the local resolution of cryo-EM density maps. *Nat Methods* **11**, 63-65 (2014).

3. Schuwirth B*, et al.* Structures of the bacterial ribosome at 3.5 Å resolution. *Science* **310**, 827-834 (2005).

4. Pulk A, Cate JH. Control of ribosomal subunit rotation by elongation factor G. *Science* **340**, 1235970 (2013).

5. Jenner LB, Demeshkina N, Yusupova G, Yusupov M. Structural aspects of messenger RNA reading frame maintenance by the ribosome. *Nat Struct Mol Biol* **17**, 555-560 (2010).

6. Ban N*, et al.* A new system for naming ribosomal proteins. *Curr Opin Struct Biol* **24**, 165-169 (2014).

7. Cannone JJ*, et al.* The comparative RNA web (CRW) site: an online database of comparative sequence and structure information for ribosomal, intron, and other RNAs. *BioMed Central Bioinformatics* **3**, 2 (2002).

8. Chiba S, Lamsa A, Pogliano K. A ribosome-nascent chain sensor of membrane protein biogenesis in *Bacillus subtilis*. *EMBO J* **28**, 3461-3475 (2009).

9. Kumazaki K*, et al.* Structural basis of Sec-independent membrane protein insertion by YidC. *Nature* **509**, 516-520 (2014).

10. Aung S*, et al.* Dual localization pathways for the engulfment proteins during Bacillus subtilis sporulation. *Mol Microbiol* **65**, 1534-1546 (2007).
